# Plasma exosomes from patients with active thyroid-associated orbitopathy induce inflammation and fibrosis in orbital fibroblasts

**DOI:** 10.1186/s12967-024-05263-y

**Published:** 2024-06-07

**Authors:** Li Wei, Qinying Huang, Yunhai Tu, Shihan Song, Xiaobo Zhang, Bo Yu, Yufen Liu, Ziwei Li, Qing Huang, Lili Chen, Bo Liu, Shenglan Xu, Tong Li, Xiyuan Liu, Xiaozhou Hu, Weijie Liu, Zai-Long Chi, Wencan Wu

**Affiliations:** 1 National Clinical Research Center for Ocular Diseases, Eye Hospital, Wenzhou Medical University, Wenzhou, 325027 China; 2 State Key Laboratory of Ophthalmology, Optometry and Vison Science, Eye Hospital, Wenzhou Medical University, Wenzhou, 325027 China; 3 Oujiang Laboratory (Zhejiang Lab for Regenerative Medicine, Vision and Brain health), Wenzhou, 32500 China

**Keywords:** Thyroid-associated orbitopathy, Exosome, miRNA, Immune response, Fibrosis

## Abstract

**Background:**

The pathogenesis of thyroid-associated orbitopathy (TAO) remains incompletely understand. The interaction between immunocytes and orbital fibroblasts (OFs) play a critical role in orbital inflammatory and fibrosis. Accumulating reports indicate that a significant portion of plasma exosomes (Pla-Exos) are derived from immune cells; however, their impact upon OFs function is unclear.

**Methods:**

OFs were primary cultured from inactive TAO patients. Exosomes isolated from plasma samples of patients with active TAO and healthy controls (HCs) were utilized for functional and RNA cargo analysis. Functional analysis in thymocyte differentiation antigen-1^+^ (Thy-1^+^) OFs measured expression of inflammatory and fibrotic markers (mRNAs and proteins) and cell activity in response to Pla-Exos. RNA cargo analysis was performed by RNA sequencing and RT-qPCR. Thy-1^+^ OFs were transfected with miR-144-3p mimics/inhibitors to evaluate its regulation of inflammation, fibrosis, and proliferation.

**Results:**

Pla-Exos derived from active TAO patients (Pla-Exos^TAO−A^) induced stronger production of inflammatory cytokines and hyaluronic acid (HA) in Thy-1^+^ OFs while inhibiting their proliferation. Kyoto Encyclopedia of Genes and Genomes (KEGG) pathway analysis and single sample gene set enrichment analysis (ssGSEA) suggested that the difference in mRNA expression levels between Pla-Exos^TAO−A^ and Pla-Exos^HC^ was closely related to immune cells. Differential expression analysis revealed that 62 upregulated and 45 downregulated miRNAs in Pla-Exos^TAO−A^, with the elevation of miR-144-3p in both Pla-Exos and PBMCs in active TAO group. KEGG analysis revealed that the target genes of differentially expressed miRNA and miR-144-3p enriched in immune-related signaling pathways. Overexpression of the miR-144-3p mimic significantly upregulated the secretion of inflammatory cytokines and HA in Thy-1^+^ OFs while inhibiting their proliferation.

**Conclusion:**

Pla-Exos derived from patients with active TAO were immune-active, which may be a long-term stimulus casual for inflammatory and fibrotic progression of TAO. Our finding suggests that Pla-Exos could be used as biomarkers or treatment targets in TAO patients.

**Supplementary Information:**

The online version contains supplementary material available at 10.1186/s12967-024-05263-y.

## Introduction

Thyroid associated orbitopathy (TAO), also known as Graves’ orbitopathy (GO), is a complex and intractable autoimmune disorder [1]. It has a high prevalence in various countries and regions of the world, with an overall incidence of 0.5%, predominantly affecting women, typically in their third to fifth decade [2]. Previous studies demonstrated that the pathophysiology of TAO involves the crosstalk of insulin-like growth factor 1 receptor with thyrotropin receptor, causing progressive orbital tissue inflammation and remodeling, ultimately resulting in substantial disfigurement and visual impairment [3, 4]. Although most cases of TAO are mild or self-limiting, up to ∼30% require treatments that are usually of an anti-inflammatory/immunosuppressive nature [1, 5]. However, these therapeutic effects remain poor or inconclusive. Hence developing an interventive measure based on the pathogenesis level of TAO is the goal of treatment, which is complicated and challenging.

Recent studies have provided extraordinary insight into the pathogenesis of TAO yet remains poorly understood, making it hard to treat effectively. Various inflammatory cells implicated in TAO activity, including T cells [6, 7], B cells [6], monocytes/macrophages [8] and mast cells [8], play an important role in maintaining orbital inflammatory environment, releasing numerous cytokines (CKs), growth factors (GFs) and other inflammatory mediators, many of which act as potent stimulators of hyaluronic acid (HA) accumulation and adipogenesis [4, 7, 8]. As orbital tissue resident cells, orbital fibroblasts (OFs) are activated to secret a variety of inflammatory mediators, thereby continuing to recruit immune cells into the orbit [7, 9]. This interaction between immunocytes and OFs maintains and exacerbates intraorbital inflammation. Inhibition of these complex immune-inflammatory processes is crucial for treatment of TAO, potentially sparing patients from undergoing multiple invasive surgeries. While chemokines and cytokines are known modulators of the immune response, exosomes have recently been identified as having a pivotal communicatory role in the initiation and resolution of inflammation [10].

Exosomes (exos), with a size range of ∼40 to 160 nm (average ∼100 nm), are a subset of extracellular vehicles (EVs) for cell-to-cell communication [11, 12]. They carry various types of cargo, including RNA molecules, proteins and lipids that reflect the cell of origin [11, 12]. Hence, the relative abundance ofdifferent exosomal molecules may reflect – potentially pathologic – changes in the relative cellular mechanisms affecting their release. Additionally, it is possible that one species of exosomal biomolecules may exhibit altered functional properties independently from another. It’s important to note that immune-related cells activation impacts exosomal composition and function, and cells exposed to such exosomes contribute to inflammation/autoimmunity, cell injury, and cellular dysfunction [11, 13]. Also, immunocyte-derived exosomes constitute a large component of peripheral blood exosomes [14]. Numerous studies have indicated that plasma exosomes (Pla-Exos) affect both proinflammatory and profibrotic processes in inflammatory/autoimmune diseases and can also alter proliferation and apoptosis in cells [15–17]. Lee et al. [15] found that exosomes in blood from patients with systemic lupus erythematosus (SLE) have the ability to stimulate peripheral blood mononuclear cells (PBMCs) to produce inflammatory cytokines. Similarly, plasma EVs induced liver cells to release inflammatory factors, leading to long-term liver fibrosis progression in patients with hepatitis C virus (HCV) [16]. Exosome miRNA-26b-3p in plasma from individuals with idiopathic short stature inhibited chondrocyte proliferation and impairs longitudinal bone growth [17]. In addition, Hiratsuka et al. [18] found that the differentially expressed circulating exosomal miRNAs were related to disease activity and involved in the regulation of immunocyte activation, playing an important role in the pathogenesis of Graves’ disease. This combined evidence suggests the need to investigate the impact of Pla-Exos on TAO development.

In this study, we assessed the influence of Pla-Exos on orbital inflammatory and fibrosis progression by analyzing the function and content of Pla-Exos. We first investigated the function of Pla-Exos derived from patients with active TAO (Pla-Exos^TAO−A^) or healthy controls (HCs)(Pla-Exos^HC^)on human OFs. Secondly, we analyzed the RNA cargo of exosomes and highlighted the essential roles of miRNA, particularly those derived from PBMCs, in exosome-mediated dysfunction of OFs. Here, we provide the first evidence that Pla-Exos^TAO−A^ are immune- and fibrotic-active for their structural modifications. Accordingly, the inflammatory process of TAO might produce “TAO-specific” exosomes that might, in return, amplify the abnormal immune response and fibrosis.

## Methods and materials

### Human samples

Blood samples were obtained from patients with active TAO (TAO-A) (*n* = 25) and healthy controls (HCs) (*n* = 25). Venous blood (4 ml) was collected in Vacutainer Plus blood collection tubes containing EDTA (BD Biosciences, San Jose, CA, USA). Plasma was obtained from blood samples via centrifugation (300 × g, 10 min, 4 °C; 3000× g, 15 min, 4 °C) to remove cell debris and large apoptotic bodies before storage at − 80 °C. Peripheral blood mononuclear cells (PBMCs) were isolated from fresh whole blood and stored at − 80 °C. Orbital connective tissues (OCTs) were obtained from inactive TAO patients (TAO-NA ) (*n* = 5) who underwent orbital decompression surgery, using automated dissociators and a 0.9% saline solution for the dissociation process, followed by preservation in phosphate-buffered saline (PBS) (Biological Industries, Kibbutz Beit Haemek, Israel) until cell extraction.

The enrolled patients in this study met the following inclusion criteria: (1) Diagnosis of TAO based on the Bartly criteria [19]; (2) aged between 18 and 70 years. The following exclusion criteria were applied: (1) A previous history of inflammatory/ autoimmune, metabolic, or neoplastic diseases; (2) treatment with glucocorticoids and/or immunosuppressive agents within the past 3 months; or (3) pregnancy or lactation in women. In all patients, 7-item Clinical activity score (CAS) [20] was used to assess TAO activity, with CAS ≥ 3 indicating active TAO. This study received the approval of the Ethics Committee of the Eye Hospital of Wenzhou Medical University, Wenzhou, China (2022-109-K-83). Written informed consent from all patients and HCs. The authors confirm that all experiments were performed in accordance with the relevant guidelines and regulations.

### Exosome isolation

Exosomes were isolated from plasma samples of active TAO patients and HCs by size exclusion chromatography (SEC) using Exosupur® columns (35 nm, Echobiotech, Beijing, China). Briefly, the plasma samples were rapidly thawed at 37℃, centrifuged at 10,000×g for 30 min at 4 °C, and processed through a 0.22 μm filter to remove large particles. Subsequently, the samples underwentfurther purification using Exosupur® columns according to the manufacturer’s instructions. The elution of the samples was carried out with PBS and a total of 2 mL eluate fractions were collected. These fractions were concentrated to 200 µL by 100 kDa molecular weight cut-off Amicon® Ultra spin filters (Merck, Germany). Exosomes were quantified using a Pierce BCA Protein Assay Kit (Thermo Fisher Scientific, Rockford, IL, USA), and the usage quantity of exosome were calculated. Equal exosomes (50 µg/mL) were added into thymocyte differentiation antigen-1^+^ (Thy-1^+^) OFs.

### Western blot

Proteins from exosomes or PBMCs were prepared as described previously [21]. Subsequently, the protein lysates were electrophresed on 10% SDS-PAGE gels. Following electrophoresis, proteins were transferred onto polyvinylidene fluoride (PVDF) membranes and blocked at room temperature in Tris-buffered saline/0.3% Tween20 containing 4% of bovine serum albumin (BSA). The membranes were then incubated overnight at 4 C with CD63 (ab68418, 1 µg/mL, Abcam, Cambridge, MA, USA), Alix (12422-1-AP, 1/1000 dilution, Proteintech, Chicago, USA), TSG101 (ab125011, 1/1000 dilution, Abcam) or Calnexin antibodies (10427-2-AP, 1/1000 dilution, Proteintech) diluted 1/1000 in 1% BSA. After washing 3 times with TBST, the membranes were incubated with horseradish peroxidase-conjugated goat anti-rabbit secondary antibody ( A0208, 1/1000 dilution, Beyotime, Shanghai, China) at room temperature for 2 h. Finally, signals were detected using a chemiluminescence kit (34,580, Thermo Fisher Scientific). The chemiluminescence reaction was visualized in iBright FL500 imaging system (Thermo Fisher Scientific).

### Nanoparticle tracking analysis (NTA)

The Vesicle suspensions with concentrations between 1 × 10^7^ /ml and 1 × 10^9^ /ml were examined using the ZetaView PMX 110 (Particle Metrix, Meerbusch, Germany) equipped with a 405 nm laser to determine the size and quantity of the particles we isolated. A video of 60-sec duration was taken with a frame rate of 30 frames/sec, and particle movement was analyzed using NTA software (ZetaView 8.02.28).

### Transmission electron microscopy (TEM)

Ten µL purified fresh exosomes were placed on a carbon-coated copper grid and incubated at room temperature for 1 min. Then the exosome was immersed in 2% uranyl acetate solution for 1 min. Excess fluid was removed using filter paper. The exosomes were observed and imaged under a transmission electron microscope (H-7650, Hitachi Ltd., Tokyo, Japan) at an accelerating voltage of 80 kV.

### PBMCs isolation

PBMCs were isolated from fresh whole blood using Ficoll-Paque density-gradient centrifugation. First, the samples were diluted at least 2-fold with PBS. Then, 3 mL of Ficoll-Paque™ PREMIUM separation solution (Cytiva, Uppsala, Sweden) was added to SEPMATE-15 (STEMCELL™ Technologies, Vancouver, Canada), followed by the addition of 5 mL of diluted blood without disrupting the upper surface of the Ficoll separation solution. Samples were centrifuged at 400 *g* for 30 min at room temperature (18–22℃). Buffy coat–containing PBMCs were transferred to a new 15-mL centrifuge tube using a 1-mL manual pipette and washed twice with PBS. Red blood cell lysis buffer (Beyotime) was added and placed on ice for 4 min before PBS was added again. The samples were centrifuged and PBMCs were eventually dissolved in freezing medium (90/10 PBS/dimethyl sulfoxide). PBMCs were stored in liquid nitrogen until needed.

### Cell preparation

Primary orbital fibroblasts were extracted from OCTs of TAO-NA patients. After removing adipose tissues and blood vessels, the OCTs were cut into 1- to 2-mm pieces, and then planted in Petri dishes. Dulbecco’s Modified Eagle Medium/Nutrient Mixture F12 (DMEM/F-12) (Biological Industries) supplemented with 20% fetal bovine serum (FBS) (HyClone, GE Healthcare, Logan, USA), 1% GlutaMAX (Gibco, New York, USA), 100 mg/mL streptomycin and 100 IU/mL penicillin (Gibco) was slowly added to cover the small tissue pieces. The orbital tissue pieces were grown in a cell incubator at 37℃ and 5% CO_2_. The medium was changed every 3 days, and OFs were obtained with 0.05% Trypsin-EDTA (Gibco) after the cells grew to confluence. The cultured strains were used between the third and sixth passages.

Thy-1^+^ OFs prepared using CD90 MicroBeads (human), LS Columns and manual MACS magnetic separator (all from Miltenyi Biotec, Bergisch Gladbach, North Rhine-Westphalia, Germany) according to the manufacturer’s instructions. Separation of pure Thy-1^+^ OFs was accomplished by 2 rounds of magnetic selection. The Thy-1^+^ subset was determined by flow cytometry. The cells were washed and resuspended in stain buffer (BD Biosciences, San Jose, CA, USA), and then incubated with allophycocyanin (APC)-conjugated mouse anti-human Thy-1 (CD90) antibody or APC-conjugated mouse IgG1 κ isotype control antibody (both from BD Biosciences, San Jose, CA, USA) for 20 min at 4 °C. After 2 washes, the cells were resuspended in 200 µL staining buffer and subjected to flow cytometric analysis (BD Canto II, Franklin Lakes, NJ, USA).

### Immunofluorescence

Thy-1^+^ OFs were seeded in 24-well plates on coverslips at approximately 2 × 10^4^ per well and cultured overnight at 37 ℃ in an atmosphere of 5% CO_2_. After washing three times with PBS, the cells were fixed with 4% paraformaldehyde for 20 min, permeabilized with PBS containing 0.2% Triton X-100 (PBST) for 30 min and blocked with 3% BSA/PBS for 30 min at room temperature. The cells were then incubated overnight with primary antibodies against fibroblast surface protein (FSP) (SAB4200821, 5 µg/mL, Sigma-Aldrich, Saint Louis, MO, USA) and vimentin (VIM) (ab137321, 1/500 dilution, Abcam) at 4°C. Then the slides were washed with PBS for 5 min again and incubated with Alexa Fluor 488 goat anti-mouse IgG or Alexa Fluor 594 goat anti-Rabbit IgG secondary antibody (Bioss, Beijing, China) at room temperature in the dark for 1 h. All the agents were applied in accordance with the manufacturer’s instructions. Nuclei were stained for 10 min with DAPI (Beyotime). After a final wash, an anti-fluorescence quenching agent (Beyotime) was added to mount the coverslips. Images were obtained using Leica DM4B (Leica Microsystems, Brønshøj, Denmark).

### Exosome labeling and tracking

Purified exosomes isolated from plasma samples of active TAO patients were labeled with PKH67 (Sigma-Aldrich) following the manufacturer’s instructions. We incubated exosomes with 500 µL Dilution C solution and 2 µl PKH67 dye in 500 µL Dilution C solution for 3 min in a dark environment. To stop the staining process, 1 mL 1% BSA/PBS was added to bind any excess dye. The exosomes were then re-extracted to remove free PKH67 dye by ultracentrifugation (100,000×g, 70 min, 4 ℃) and subsequently added to unstained Thy-1^+^ OFs (10^4^ cells per well in 24-well plates) to evaluate exosome uptake. Following incubation for 12 h at 37 °C, the cells were fixed and stained with DAPI for 10 min at room temperature. Cells were observed and imaged using a confocal microscope (Leica DM4b, Wetzlar, Germany).

### Cell culture and treatment

To ensure consistent initial conditions for all experiments, we added 20% FBS to the medium when we started cultures from tissues or from frozen cell stocks. In addition, we used 10% FBS when pretreating the cells to ensure normal growth. To synchronize the cells in the same cell cycle phase, we replaced the culture medium with a medium containing 1% FBS before the stimulation with exosomes. Thy-1^+^ OFs were incubated with exosomes (50 µg/mL) isolated from plasma samples of HCs and active TAO patients in a medium containing 10% exosome-depleted FBS (System Biosciences, Exo-FBS-50 A-1, USA) at 37℃ in a humidified 5% CO_2_ atmosphere for 3 days (mRNA detection) and 4 days (protein detection). miR-144-3p mimics (100 nM) or inhibitors (200 nM) were transfected into Thy-1^+^ OFs using attractene transfection reagent (QIAGEN, Frederick, MD, Germany) per manufacturer’s protocol. Two days after transfection, the cells were harvested with TRIzol for RNA analysis. Three days after transfection, the supernatant was collected in tubes for protein analysis. MiR-144-3p mimics/inhibitors, control mimics/inhibitors were purchased from Guangzhou RiboBio Co., Ltd. (China), and their sequences are listed in Table S1.

### RNA isolation and RT-qPCR

Total RNA was extracted from cells using TRIzol reagent (Beyotime) and from exosomes (derived from 500 µL plasma) using RNeasy Mini Kit (QIAGEN) according to the manufacturer’s instructions. RNA concentration and purity were quantified using a Multiskan Mk3 System (Thermo Fisher Scientific). Reverse transcription of 300 ng RNA into cDNA was performed using the HiScript®III RT SuprtMix for qPCR with gDNA wiper (Vazyme, Nanjing, China), followed by amplification using the Applied Biosystems Veriti PCR system (Thermo Fisher Scientific). RT-qPCR was performed with Taq Pro Universal SYBR qPCR Master Mix (Vazyme) using QuantStudio™ 3 Real-Time PCR System (Thermo Fisher Scientific). Delta delta CT method was used to calculate genes/miRNAs expression. Gene expression results was normalized to the expression level of GAPDH; exosomal miRNA expression results were normalized to the expression level of miR-33a-5p; cell miRNA expression results were normalized to the expression level of U6. All primers are synthesized by Guangzhou RiboBio Co., Ltd., and RT primers and qPCR primers are listed in Table S2 and Table S3.

### High-throughput sequencing of exosomal RNA

Total RNA in exosomes extracted from plasma samples in the HC and TAO-A groups (*n* = 5 for each group) was sent to EchoBiotech Co. Ltd (Beijing, China) for whole transcriptome sequencing. The QIAseq miRNA Library Kit (QIAGEN) and SMARTer Stranded Total RNA-Seq Kit v2 (TAKARA, San Jose, CA, USA) were used to create miRNA and lncRNA libraries respectively, and the quality of the libraries was assessed on the Bioanalyzer 2100 system (Agilent Technologies, Santa Clara, CA, USA) and qPCR. Finally, the quantified libraries were sequenced using an Illumina NovaSeq 6000 platform (San Diego, CA, USA).

### Bioinformatics analysis

Quality control was performed on raw reads using Fastp (version 1.0), and clean reads were obtained by aligning with Silva database, GtRNAdb database, Rfam database and Repbase database to filter the low-quality data. Then, HISAT2 (version 2.2.0), Stringtie (version 1.3.6), and miRDeep2 (version 0.1.3) software were respectively used to map and annotate mRNAs, lncRNAs, and miRNAs based on the Human Genome (version GRCm38) and miRBase. Afterward, the RNA expression levels from the 10 samples were first transformed on a log_2_ scale before principal component analysis (PCA). PCA was performed using the prcomp function in stats package in R3.6.1. Using edgeR package (version 3.40.0) in R3.6.1, differentially expressed lncRNAs (DE-lncRNAs), miRNAs (DE-miRNAs) and mRNAs (DE-mRNAs) were screened at the thresholds of fold change > 1.5 and *p* < 0.05. Hierarchical clustering analysis was applied to depict the expression pattern of different transcripts using Pheatmap package (version 1.0.8) in R3.6.1. To visualize volcano plots, we utilized the plot function in the graphics package in R. We performed in silico analysis to identify putative miRNA target genes using TargetScan, miRanda, PicTar, MicroCosm Targets and miRDB; the genes predicted in at least three sites were identified as the target genes of DE-mRNAs. Subsequently, functional and pathway enrichment analyses were performed using Kyoto Encyclopedia of Genes and Genomes (KEGG) pathway, and the enrichment score generated by -log10 (p-value) / *P* < 0.05 depicted the importance of the pathway correlations. Single-sample gene-set enrichment analysis (ssGSEA) on DE-mRNAs was carried out by GSVA package (version 1.48.3) in R3.6.1, and the normalized ssGSEA score was calculated to evaluate the enrichment levels of immunocytes in each sample [22]. In addition, miRcode was used to predict miRNA binding sites to predict the interactions between miRNA and its target genes, and the nodes are linked with enriched genes. Table S4 gives an overview about several databases available.

### Enzyme-linked immunosorbent assay (ELISA)

The concentrations of IL-1β, IL-6, TNF-α, CXCL2, and RANTES and HA in the cell culture supernatant were quantified with ELISA kits (R&D Systems, Minneapolis, MN, USA) according to the manufacturer’s instructions.

### Cell counting kit-8 (CCK-8) assay

Cell proliferation was evaluated by CCK-8 assay. Thy-1^+^ OFs were inoculated into 96-well cell culture plates at 3000 cells per well in complete medium, then treated with exosome or miR-144-3p mimics/inhibitors. Ten µl CCK-8 solution (Beyotime) was added to each well, and cells were incubated again for 1.5 h. The absorbance at 450 nm was measured using a microplate reader (Thermo Multiskan MK3, USA) at 0 h, 24 h, 48 h, 72 h, or 96 h.

### Statistical analysis

Statistical analyses were performed using GraphPad Prism (vision 9.3.5). Student’s *t*-test was used to compare two groups, and one-way ANOVA for multiple group comparison. All the data were presented as mean ± standard deviation (SD). Differences were considered significant when *p* < 0.05.

## Results

### Study design and patient characteristics

**Fig. 1:**
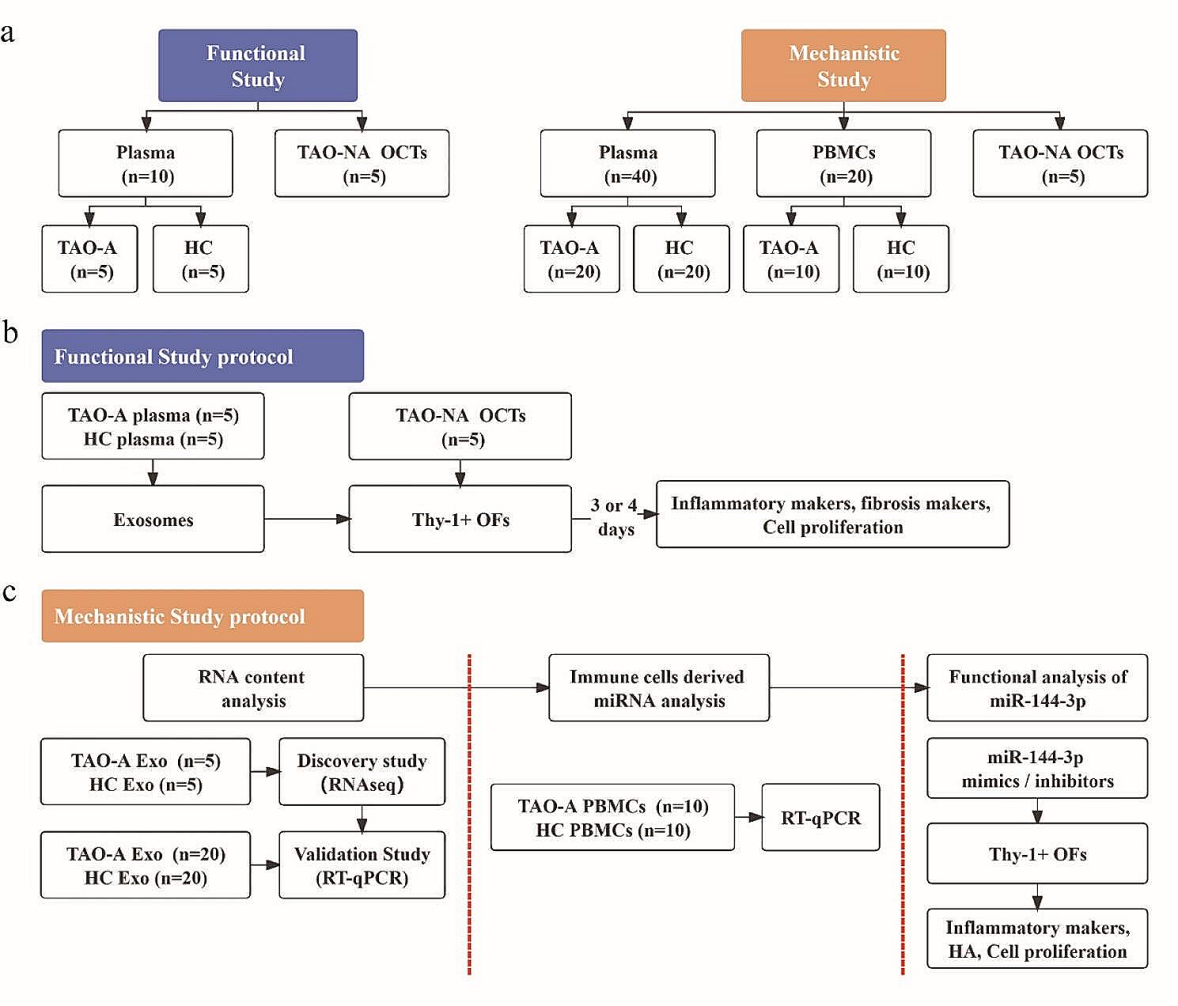
Design of the study. **(a)** An overall workflow of the entire study. **(b)** The protocol used in the functional study. After adding exosomes 3 days (mRNA detection) or 4 days (protein detection), the inflammatory markers, fibrosis markers, and cell proliferation in Thy-1^+^ OFs were assessed. **(c)** The protocol used in the mechanistic study of exosomes. Exosomal RNA content was analyzed using RNA-seq and RT-qPCR. Immune cell-derived miRNA was selected using RT-qPCR. miR-144-3p was selected as an immunocyte-derived miRNA and miR-144-3p mimics/inhibitors were transfected into Thy-1^+^ OFs to evaluate its regulation of inflammation, fibrosis, and cell proliferation. TAO-A active TAO patients, HCs healthy controls, TAO-NA inactive TAO patients, OCT orbital connective tissue, PBMCs peripheral blood mono-nuclear cells, OFs orbital fibroblasts, RNA-seq RNA sequencing, HA hyaluronic acid

As demonstrated by the overall workflow (Fig. 1a), the study consisted of two parts: a functional study and a mechanistic study. Functional analysis of exosomes was performed on Thy-1^+^ OFs (Fig. 1b). Exosomes were obtained from plasma samples of patients with active TAO (*n* = 5) and HCs (*n* = 5), and the OFs were separated from orbital connective tissues (OCTs) of patients with inactive TAO (TAO-NA) (*n* = 5) who underwent orbital decompression surgery. Mechanistic analysis was performed using RNA content (Fig. 1c). First, we performed a discovery study for RNA expression profile using whole transcriptome high-throughput sequencing analysis and analyzed the differentially expressed RNAs in exosomes from the same plasma samples as described above. Next, another series of plasma samples were collected from patients with active TAO (*n* = 20) and HCs (*n* = 20) to validate the exosome miRNA expression profile. The miR-144-3p level was evaluated in both Pla-Exos and PBMCs, and the network and functional analysis were made to show its potential function. Finally, miR-144-3p was screened as a potential functional biomolecule for functional analysis. The clinical characteristics of the patient populations studied are shown in Table S5.

### Plasma exosomes were taken up into OFs

**Fig. 2:**
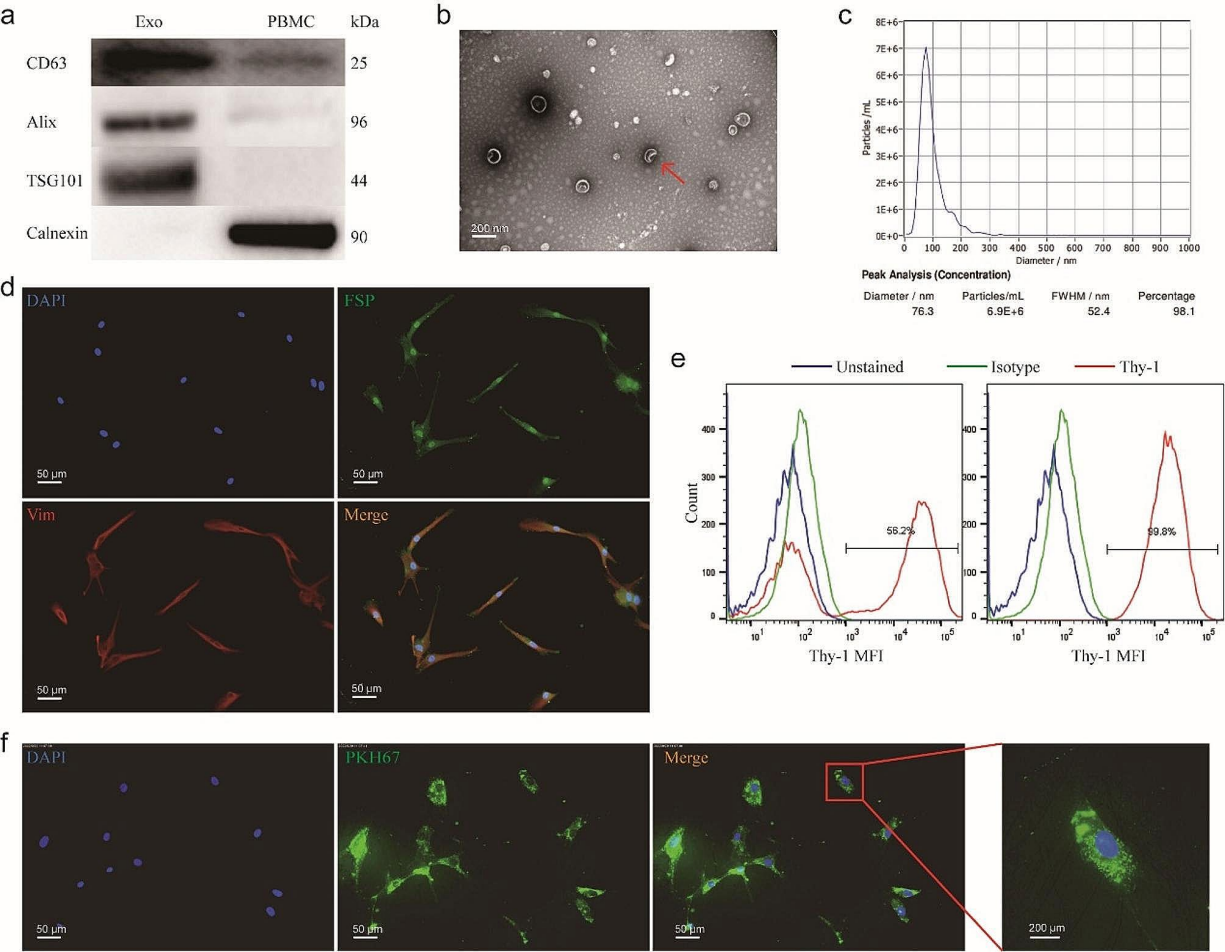
Plasma exosomes were taken up by human OFs. **(a-c)** Characterization of Pla-Exos. **(a)** Western blot analysis identified positive expression of exosomes markers CD63, Alix and TSG101, with the negative expression of Golgi marker Calnexin in the extraction samples. **(b)** Nano-Sight analysis indicated that the size of the extraction was approximately 100 nm. **(c)** The morphology of the extraction was examined via transmission electron microscope. **(d, e)** Characterization of OFs. **(d)** Immunofluorescence staining of OFs reveals the uniform expression of the fibroblast markers FSP and vimentin. Cells were stained with anti-FSP and anti-vimentin antibodies and Alexa Fluor 488/594-conjugated secondary antibodies (fluorescence microscopy, 20; scale bars, 50 μm). **(e)** OFs were analyzed for Thy-1 expression using flow cytometry (right). The Thy-1^+^ subset was obtained from parental OFs after 2 rounds of magnetic bead sorting. Thy-1^+^ OFs were > 99% positive for Thy-1 (left). **(f)** Pla-Exos (green) were transferred into OFs (blue) (fluorescence microscopy, 20). FSP fibroblast surface protein, VIM vimentin

Exosomes were purified from plasma samples of patients with active TAO and HCs using SEC and identified using western blotting, NTA and TEM (Fig. 2a – c). Western blotting results suggested that the vesicles positively expressed the exosome protein markers CD63, Alix, and TSG101, and were negative for the Golgi marker – Calnexin (Fig. 2a). TEM showed that the exosomes were cup-shaped or round, with a diameter of approximately 100 nm (Fig. 2b). Additionally, NTA revealed most exosomes to be predominantly in the 50–150 nm size range (Fig. 2c). No significant differences in exosome concentrations existed between the two groups (Fig. S1). Hence, the vesicles we isolated were confirmed to be exosomes.

Thy-1^+^ OFs were separated from the OCTs of patients with inactive TAO and examined by immunofluorescence assays and flow cytometry (Fig. 2d, e). The oblong, spindle-shaped cells were morphologically consistent with the fibroblast phenotype, and they grew in adherent monolayers to plastic substratum. These fibroblasts maintained a stable phenotype over the indicated culture interval and uniformly expressed fibroblast surface protein 1 (FSP-1) and vimentin (Fig. 2d). Thy-1 (CD90) expression in OFs was evaluated by flow cytometry. The Thy-1^+^ subset was isolated from parental OFs via 2 rounds of magnetic bead selection. Thy-1^+^ fibroblasts were > 99% positive for Thy-1 (Fig. 2e). All these indicated that the cultured cells were Thy-1^+^ OFs.

To confirm that exosomes could be transferred into Thy-1^+^ OFs, we labeled Pla-Exos with a fluorescent membrane marker PKH67. The PKH67-labeled Pla-Exos were incubated with unstained Thy-1 ^+^ OFs for 12 h. Confocal imaging revealed the delivery of labeled exosomes, as indicated by the presence of fluorescent membrane dyes in unlabeled OFs (Fig. 2f).

### Plasma exosomes derived from patients with active TAO promotes Thy-1^+^ OFs inflammation and fibrosis

**Fig. 3:**
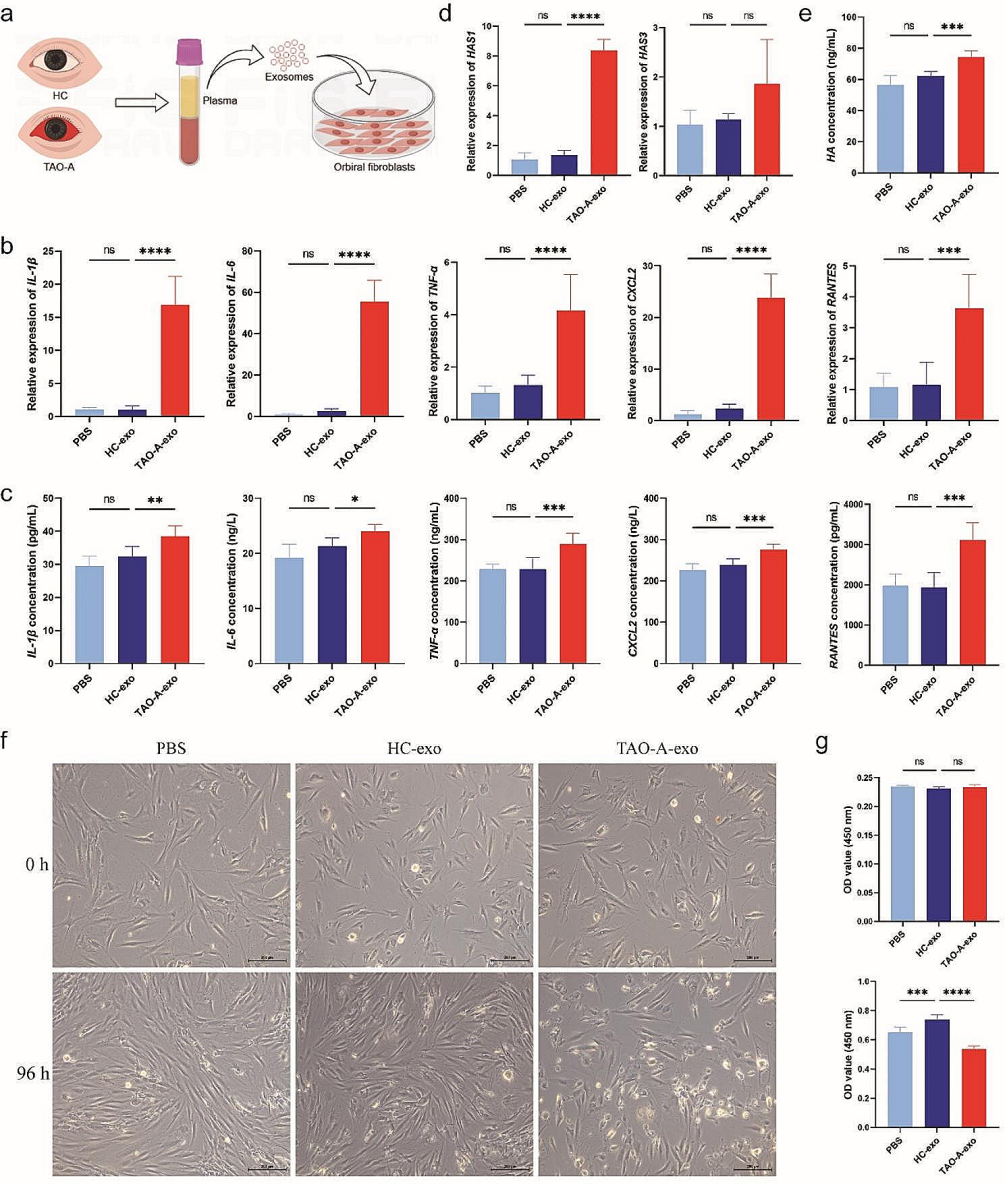
Plasma exosomes derived from patients with active TAO induced stronger production of cytokines and HA in Thy-1^+^ OFs and inhibited their proliferation. **(a)** Diagram of the Pla-Exos treating Thy-1^+^ OFs. Pla-Exos (50 µg/mL) derived from 5 HCs and 5 patients with active TAO were added to Thy-1^+^ OFs. mRNA and protein detection were performed after 72–96 h, respectively. **(b, c)** Pla-Exos^TAO−A^ induced stronger production of inflammatory molecules in Thy-1^+^ OFs. The mRNA levels of IL-1β, IL-6, TNF-a, CXCL2 and RANTES determined by RT-PCR and normalized to GAPDH mRNA levels are shown in **(b)**. The concentrations of IL-1β, IL-6, TNF-a, CXCL2 and RANTES in cell supernatants detected by ELISA are shown in **(c)**. **(d, e)** Pla-Exos^TAO−A^ induced stronger production of HA in Thy-1^+^ OFs. The mRNA levels of HAS1 are shown in (d). The concentrations of HA in cell supernatants are shown in **(e)**. **(f, g)** Pla-Exos^TAO−A^ inhibited the proliferation of Thy-1^+^ OFs. Data are representative of 3 independent experiments. *n* = 6 in each group. Error bars represent the mean ± SD. ns, no significance, *p < 0.05, **p < 0.01, ***p < 0.001, ****P ≤ 0.0001, one-way ANOVA with Dunnett’s comparison test. HA Hyaluronic acid, HAS hyaluronan synthase, SD standard deviation

To investigate whether Pla-Exos acts as an agonist or antagonist in TAO development, Thy-1^+^ OFs were pretreated with Pla-Exos derived from patients with active TAO or HCs (Fig. 3a). As shown in Fig. 3b and c, Pla-Exos^TAO−A^ induced higher levels of inflammatory molecules (IL-1β, IL-6, TNF-α, CXCL2 and RANTES) than Pla-Exos^HC^-treated and untreated Thy-1^+^ OFs. Notably, the mRNA level of the fibrogenic marker hyaluronan synthase (HAS) 1 was significantly higher in Thy-1^+^ OFs treated with Pla-Exos^TAO−A^ (Fig. 3d), and this observation was confirmed at the protein level for HA (Fig. 3e); however, Pla-Exos^HC^ didn’t share this ability. Excessive HA production is one of the main components responsible for orbital tissue expansion and fibrosis in TAO. Besides, neither TAO-A- nor HC-derived Pla-Exos stimulated the secretion of α-smooth muscle actin (a-SMA) and Collagen in Thy-1^+^ OFs (Fig. S2b). Finally, a dramatic reduction in the proliferation of Thy-1^+^ OFs was observed under Pla-Exos^TAO−A^ stimulation (*P <* 0.0001), while this feature wasn’t observed in Thy-1^+^ OFs treated with Pla-Exos^HC^ (Fig. 3f, g). These data provide evidence for a plasma exosome-mediated inflammatory and fibrogenic stimulus in patients with active TAO. In contrast, plasma exosomes derived from patients with active TAO inhibited cell viability of Thy-1^+^ OFs compared with those derived from HCs.

### RNA signature of plasma exosomes derived from patients with active TAO

To correlate functional properties with structural evidence, we further analyzed the RNA cargo in Pla-Exos isolated from the same patients with active TAO and HCs as described above. We investigated the RNA expression profiles of Pla-Exos by RNA sequencing (RNA-seq) and used a 1.5-fold change and *p* value < 0.05 as the threshold to define differentially expressed RNAs.

**Fig. 4:**
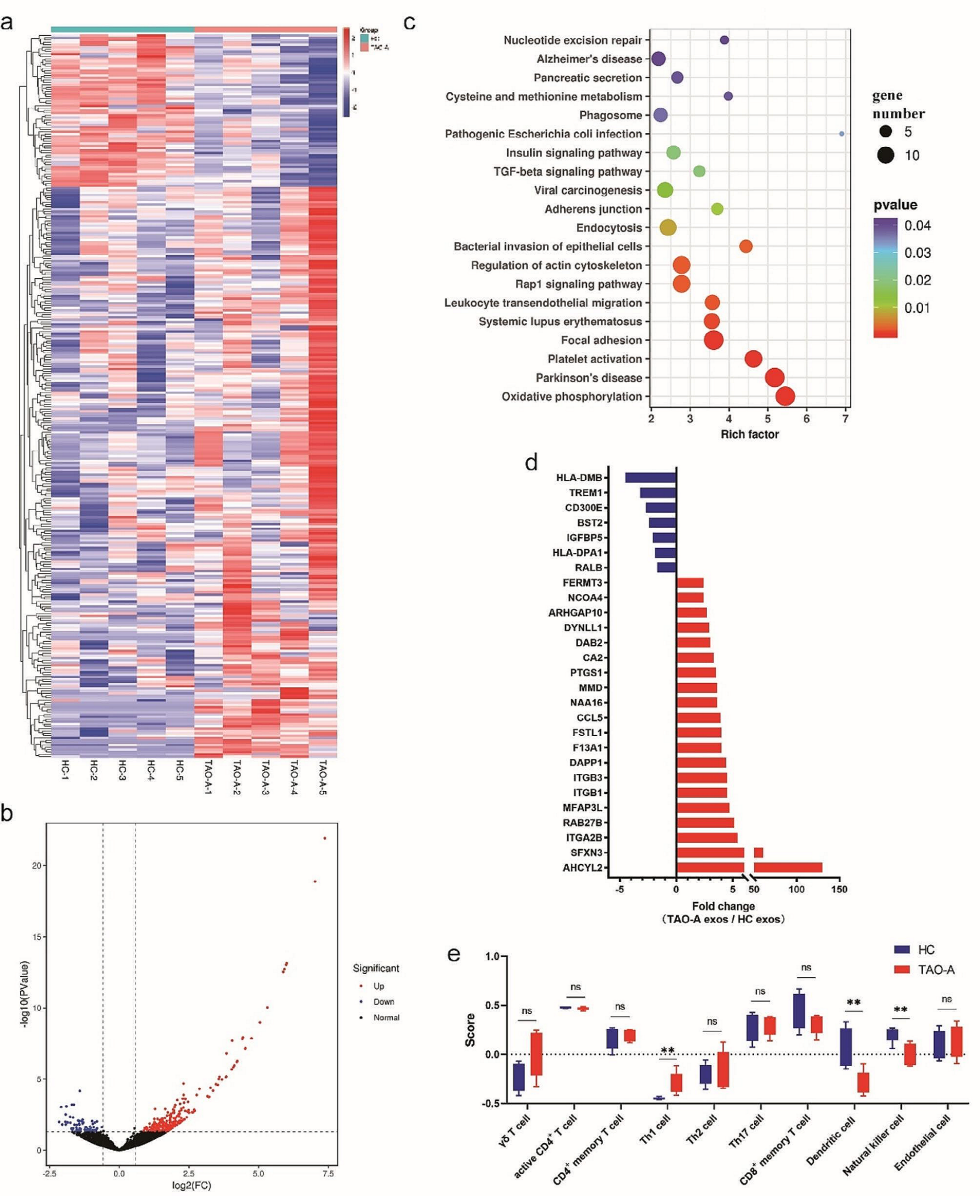
The exosomal mRNA profiles of patients with active TAO differed from those of healthy controls. **(a)** Heatmap of the mRNA expression profiles in the TAO-A and HC groups. Exosomes were isolated from the same plasma samples used in the functional analysis. RNA-seq was used for mRNA content analysis. **(b)** Volcano plot comparing mRNAs in Pla-Exos from patients with active TAO and HCs. The volcano plot was created using a log2 fold change and –log10 *p* values of all the detected mRNAs. **(c)** Results of KEGG pathway analysis for the DE-mRNA. **(d)** The differentially expressed immune cells marker genes in exosomes were examined. **(e)** Enrichment levels of immune subpopulations in Pla-Exos. Th1 cell marker genes increased, while dendritic cell and natural killer cell marker genes decreased in Pla-Exos of active TAO patients. RNA-seq RNA sequencing, KEGG Kyoto Encyclopedia of Genes and Genomes analysis, DE-mRNA differential expressed mRNA.

With respect to mRNA cargo, 243 upregulated mRNAs and 65 downregulated mRNAs were found in Pla-Exos^TAO−A^ compared with Pla-Exos^HC^ (Fig. 4a, b). KEGG analysis revealed that the DE-mRNAs were enriched in the immune-related signaling pathways, such as TGF-β signaling pathway and leukocyte transendothelial migration (Fig. 4c). Among the DE-mRNAs, we identified 27 categories of immune-related maker genes (Fig. 4d, Table S6). Considering that exosomes react to the functional state of the source cell, we explored the relationship between exosome-mRNAs and immune-related cells. ssGSEA was carried out to evaluate the enrichment levels of immunocytes in each Pla-Exos sample. We estimated 29 immune-related cells including 13 innate immunocytes (activated dendritic cells (DCs), CD56^bright^ natural killer (NK) cells, CD56^dim^ NK cells, eosinophils, immature DCs, macrophages, mast cells, myeloid-derived suppressor cells, monocytes, NK cells, natural killer T cells, neutrophils, and plasmacytoid DCs), 15 adaptive immunocytes (activated B cells, activated CD4 T cells, activated CD8 T cells, central memory CD4 T cells, central memory CD8 T cells, effector memory CD4 T cells, effector memory CD8 T cells, γδT cells, immature B cells, memory B cells, regulatory T cells, T follicular helper cells, Th1 cells, Th17 cells, and Th2 cells), and endothelial cell, with their marker genes were obtained from previous studies [23, 24], as shown in Table S7. Unsurprisingly, the results of ssGSEA revealed that the immune signatures of Th1 cells increased, and the immune signatures of NK cells and DCs decreased (Fig. 4e). Consistent with the functional analysis on Thy-1^+^ OFs, Pla-Exos-mRNA correlated with a significant difference between HCs and active TAO patients, thus indicating that exosome-mediated signals still contained inflammatory informational content. These results also provide clues to the functional differences conferring to immune origin of exosomes.

**Fig. 5:**
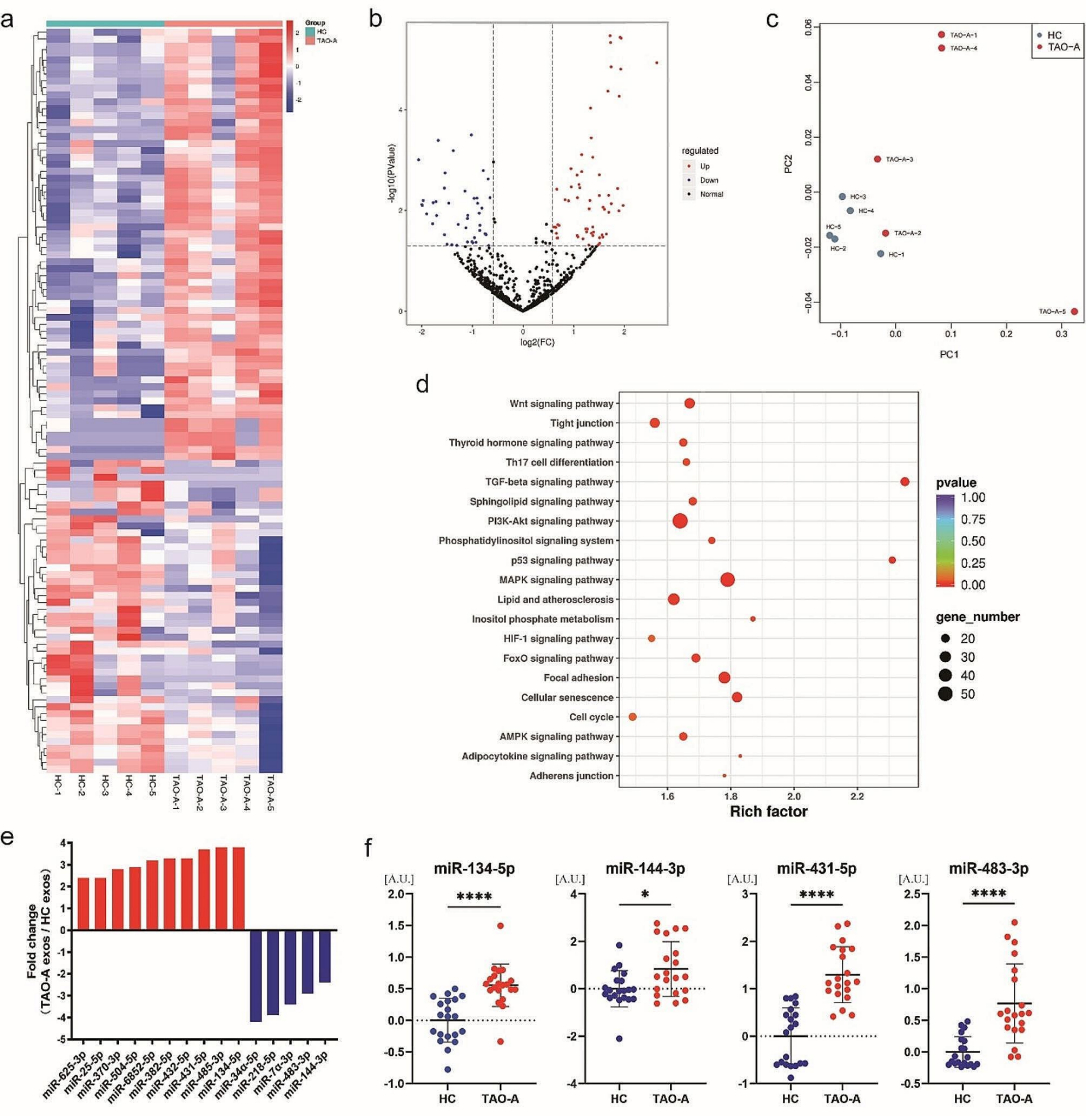
The exosomal miRNA profiles of patients with active TAO differed from those of healthy controls. **(a)** Heatmap of the miRNA expression profiles in Pla-Exos in HC and TAO-A groups. Exosomes were isolated from the same plasma samples used in the functional analysis. RNA-seq was used for miRNA content analysis. **(b)** Volcano plot comparing miRNAs in Pla-Exos from patients with active TAO and HCs. The volcano plot was created using a log2 fold change and –log10 *p* values of all the detected miRNAs. **(c)** PC analysis for the active TAO patients and HC samples based on the miRNA expression profiles. **(d)** KEGG pathway analysis of DE-miRNA target genes identified 20 immune-related pathways. **(e)** The differently expressed trends of candidate miRNAs in RNA-seq data. **(f)** Levels of miR-134-5p, miR-144-3p, miR-431-5p, and miR-483-3p in Pla-Exos determined by RT-PCR and normalized to the expression levels of miR-30a-5p. *n* = 20 per group. PC principal component, KEGG Kyoto Encyclopedia of Genes and Genomes analysis, DE-miRNA differential expressed miRNA, A.U. arbitrary unit

With respect to miRNA cargo, no significant difference was found in the count of miRNA reads or the overall expression of these miRNAs in patients with active TAO and HCs (data not shown). However, when we analyzed the expression spectrum of individual miRNAs in Pla-Exos derived from patients with active TAO and HCs, we found that there were distinct profiles of expression (Fig. 5c), indicating the potential value of the exosome miRNA analysis for the diagnosis or treatment of active TAO. To further assess the differences between the groups, we performed a cluster analysis of differentially expressed miRNAs which highlighted 62 upregulated and 45 downregulated miRNAs in active TAO patients compared with HCs (Fig. 5a, b). As revealed by KEGG pathway analysis, the confirmed target genes of the DE-miRNAs were highly enriched in the immune-related signaling pathways (Fig. 5d). To confirm the DE-miRNAs, miRNA expression levels per equal volume of Pla-Exos were determined using RT-qPCR. We selected 15 DE-miRNAs that were coherently expressed at the same level in all ten samples for analysis (Fig. 5e; Table 1). The RT-qPCR analysis required the identification of stable miRNAs for use as reference factors. Consistent with previous research [25], miR-33a-5p was found to have stable expression levels in both groups (Fig. S3a) and was therefore used as reference miRNA for normalization. Notably, expression levels of miR-134-5p, miR-144-3p, miR-431-5p, and miR-483-3p were increased in Pla-Exos^TAO−A^ compared to Pla-Exos^HC^ (Fig. 5f). This analysis is conceivably causally associated with the functional observation previously described, since both bioinformatic predictions and previous reports pinpoint many of these DE-miRNAs as regulators of inflammatory and fibrotic markers.

**Table 1:** Differentially expressed miRNAs list in patients with active TAO vs. HC

Name	Fold change	*p* value	FDR
hsa-miR-34α-5p	-4.2^†^	0.000	0.069
hsa-miR-218-5p	-3.9	0.006	0.182
hsa-miR-7α-3p	-3.4	0.007	0.185
hsa-miR-483-3p	-2.9	0.004	0144
hsa-miR-144-3p	-2.4	0.019	0.333
hsa-miR-134-5p	3.8	0.000	0.001
hsa-miR-432-5p	3.3	0.000	0.001
hsa-miR-25-5p	2.4	0.023	0.360
hsa-miR-382-5p	3.3	0.000	0.002
hsa-miR-485-3p	3.8	0.000	0.003
hsa-miR-6852-5p	3.2	0.005	0.176
hsa-miR-504-5p	2.9	0.044	0.480
hsa-miR-431-5p	3.7	0.000	0.007
hsa-miR-370-3p	2.8	0.005	0.176
hsa-miR-625-3p	2.2	0.000	0.062

^†^ “-” indicates that exosomal miRNA expression is down-regulated in the TAO-A group compared with the HC group

Only 23 lncRNAs were differentially expressed in Pla-Exos between HC and TAO-A groups, and almost all of them had low or unstable expression levels (Fig. S4). Therefore, further research was not conducted on them.

Overall, our data highlight a correlation between the function and the RNA contents of exosomes, implicating Pla-Exos as a relevant player in active TAO-related pathogenesis. Our data shows a few inflammatory modifications that act as a warning for the progression of long-term inflammatory response based on exosomes. We expected that monitoring the expression levels of these exosome-RNAs might prove to be useful for distinguishing active TAO and evaluating the clinical activity.

### MiR-144-3p expression is increased in PBMCs from patients with active TAO

**Fig. 6:**
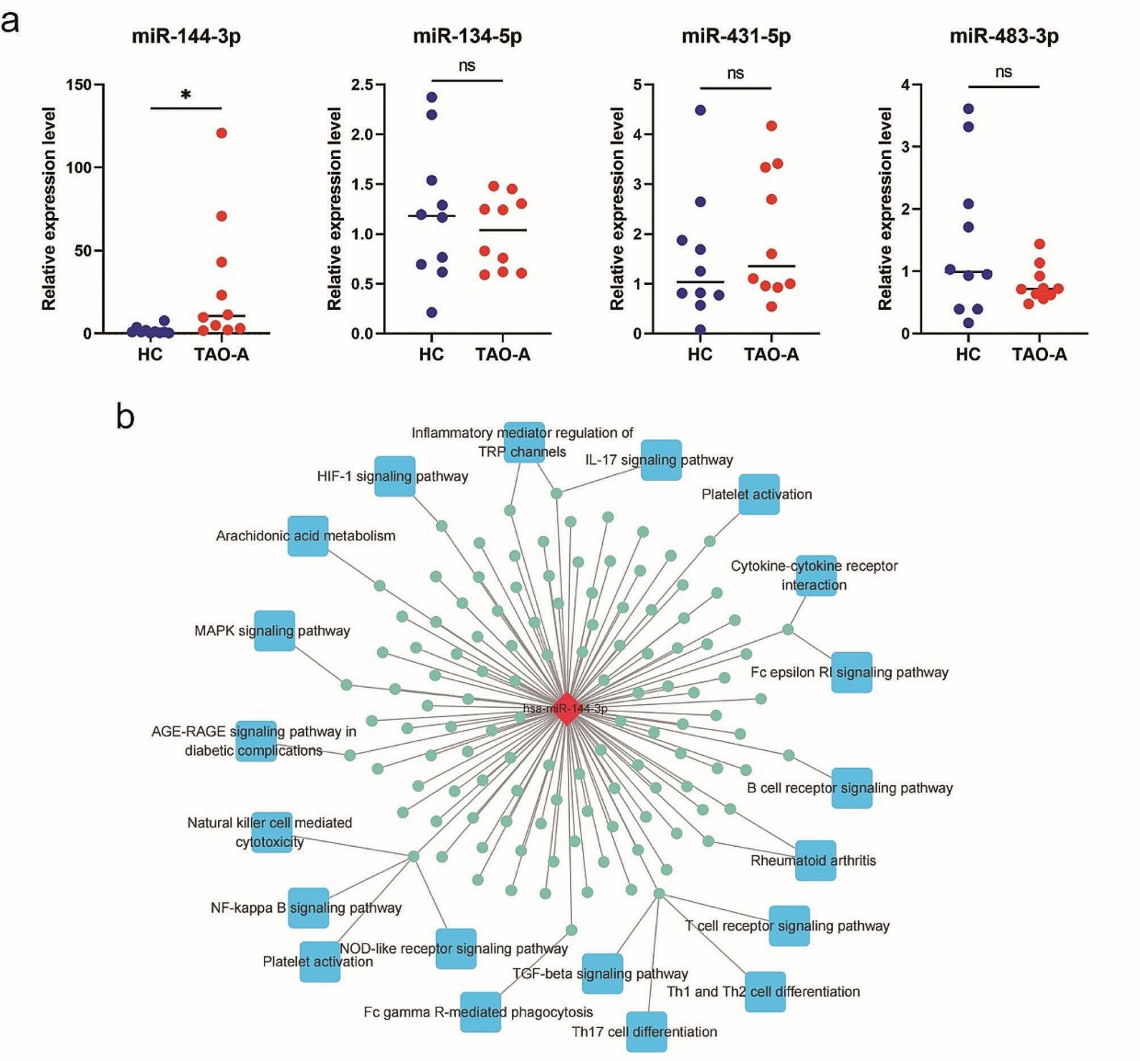
miR-144-3p expression was increased in PBMCs from patients with active TAO. **(a)** Levels of miR-134-5p, miR-144-3p, miR-431-5p and miR-483-3p in PBMCs determined by RT-PCR and normalized to the U6 expression levels. **(b)** miR-144-3p-target gene pathway network. Error bars represent the mean ± SD. ns no significance, * *p* < 0.05, student’s *t* test. PBMCs peripheral blood mononuclear cells

Activated circulating immune cells, particularly T cells, play a crucial role in the progression of TAO [26]. Exosomes are known to contain similar contents and functions as their parent cells, attributed to the presence of a distinct signature of nucleic acids, proteins, and lipids that mirrors their cellular and tissue origin. A previous study has shown that exosomes derived from immune cells constitute a significant portion of Pla-exos [14]. In this context, miRNA in Pla-Exos may serve as a tool for communication from activated leukocytes to OFs; therefore, the expression levels of miR-134-5p, miR-144-3p, miR-431-5p and miR-483-3p were further evaluated in PBMCs to identify functional miRNAs originating from immune cells. Compared with PBMCs derived from HCs, expression of miR-144-3p was elevated in PBMCs derived from patients with active TAO (Fig. 6a). The network of miR-144-3p and target genes was made to show its potential function, revealing that many target genes of miR-144-3p enriched in immune-related signaling pathways (Fig. 6b). Subsequently, we selected miR-144-3p for further analysis because miR-144-3p were upregulated in both Pla-Exos and PBMCs of active TAO patients (Figs. 5f and 6a), which suggested that miR-144-3p may be a specific immunocyte-derived exosome-miRNA that could be responsible for exosome-induced Thy-1^+^ OFs dysfunction.

### MiR‑144-3p induced proinflammatory cytokines and HA production in Thy-1^+^ OFs

**Fig. 7:**
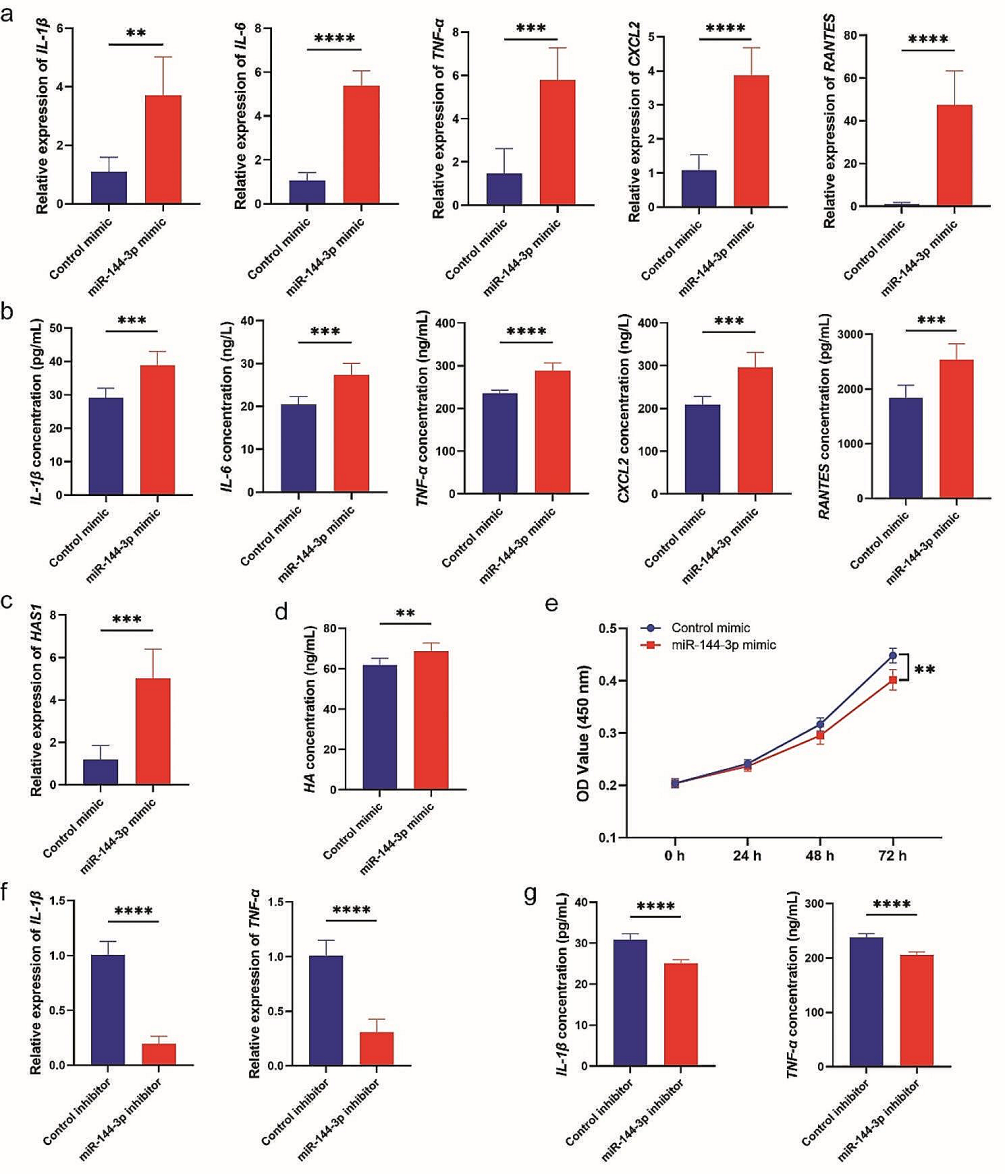
miR-144-3p induced Thy-1^+^ OFs dysfunction. **(a, b)** Levels of inflammatory molecules in Thy-1^+^ OFs treated with miR-144-3p mimics or control mimics. **(c, d)** Levels of HAS and HA in Thy-1^+^ OFs treated with miR-144-3p mimics or control mimics. **(e)** Cell proliferation of Thy-1^+^ OFs treated with miR-144-3p mimics or control mimics. **(f, g)** Levels of inflammatory molecules in Thy-1^+^ OFs treated with miR-144-3p inhibitors or control inhibitors. Error bars represent the mean ± SD. *n* = 6 per group. ns no significance, * *p* < 0.05, ** *p* < 0.01, *** *p* < 0.001 and **** *p* < 0.0001, student’s *t* test

To formally acquire mechanistic insights on miR-144-3p-specific role in OF activation and proliferation, a miRNA mimic-based analysis was performed. The miR-144-3p mimic and control mimic were transfected into Thy-1^+^ OFs, and the potential direct/indirect target mRNAs (inflammatory molecules, HAS1, HAS2, HA) and cell proliferation were quantified. As shown in Fig. 7, this analysis provided functional evidence recapitulating the exosome-based observations. The results were causally associated with the functional observations previously described, since previous reports have pinpointed miR-144-3p as a regulator of inflammatory makers [27], fibrotic markers [28], and cell proliferation [29]. Consistent with the functional observations, treatment with miR-144-3p mimic significantly augmented the expression of inflammatory molecules (IL-1β, IL-6, TNF-α, CXCL2, RANTES) (Fig. 7a, b) and increased the levels of HA-associated proteins (HAS1, HA) (Fig. 7c, d). Compared to the control mimics, the proliferative ability of Thy-1^+^ OFs was also inhibited by miR-144-3p mimics (Fig. 7e). Importantly, miR-144-3p inhibitors eliminated the antiproliferation, proinflammatory and profibrogenic effects in Thy-1^+^ OFs (Fig. 7f, g and S5). Taken together, these data support the concept that treatment with the miR-144-3p mimic significantly induces stronger cytokine, HA production and attenuates cell proliferation in Thy-1^+^ OFs.

## Discussion

The major outcome of this study provides evidence of the impact of plasma exosomes derived from patients with active TAO on orbital fibroblast activation. The comparison between Pla-Exos^TAO−A^ and Pla-Exos^HC^ highlights the functional differences correlating with exosome structural properties. Functionally, Pla-Exos^TAO−A^ displays a proinflammatory and profibrotic ability (i.e., decrease of Thy-1^+^ OFs pro-inflammatory and fibrogenic markers) that is lost in Pla-Exos^HC^; Pla-Exos^TAO−A^ inhibits Thy-1^+^ OFs proliferation. These functional differences correlate with specific cargo signatures in terms of miRNAs and mRNAs.

We provide the first evidence that active TAO-derived Pla-Exos can activate Thy-1^+^ OFs to induce the expression of proinflammatory and fibrogenic markers. In addition, they were also found to inhibit proliferation in Thy-1^+^ OFs. Exposure to Pla-Exos^TAO−A^ significantly upregulated the expression and secretion levels of proinflammatory factors (e.g., IL-1β、IL-6、TNF-ɑ、CXCL2 and RANTES) in Thy-1^+^ OFs compared with Pla-Exos^HC^. As a major inflammatory and fibrotic mediators, the upregulation of these cytokines and chemokines promotes immune cell recruitment to the orbital connective tissues [30–32] and enhances HA accumulation in the extracellular matrix [8, 33, 34], which may be an important cause of constant inflammation and fibrosis in TAO. Similarly, previous studies have confirmed that circulating exosomes derived from patients with Graves’ disease [35] or active SLE [15] have immunological activity to induce PBMCs to secrete more inflammatory cytokines [15]. Additionally, Hijmans et al. [36] also identified that HIV patients derived Pla-Exos induced proinflammatory immune response and apoptosis of endothelial cells, which may be related to the increased incidence of cardiovascular diseases in patients. Critically, a recent study on HCV highlighted that fibrogenic signals persist in EVs derived from plasma and are conceivably causal for long-term liver disease progression in patients with HCV [16]. Hence, our data suggest that Pla-Exos^TAO−A^ has proinflammatory activity, thereby participating in the immunologic derangement and fibrosis process of TAO.

Exosomes are known to have similar contents and functions as their source cells, because exosomes contain a unique signature of nucleic acids, proteins and lipids, reflecting their cell and tissue of origin [11, 13, 37]. With respect to exosome mRNA cargo, the ssGSEA results showed that Th1 cell marker genes were upregulated in Pla-Exos^TAO−A^, consistent with a systematic review study conducted by Fang, et al., in which they suggested that Th1 immune response predominates in the TAO autoimmunity, especially in early active phase [7]. At the same time, the marker genes of DC and NK cells were found to downregulate in Pla-Exos^TAO−A^. A previous study showed that mDCs are negatively related to TAO incidence [38]. In inflammatory/autoimmune diseases, immune cells (e.g., T cells, B cells, and monocytes) activate and release inflammatory exosomes, and these inflammatory exosomes also play a key role in physiological and pathological processes, such as immune response, antigen presentation, and immune cell differentiation [10, 39, 40]. Circulating T cells abnormally activate in active TAO and are associated with TAO activity [26, 41, 42]. Hence, T cells-derived exosomes in patients with active TAO may also be immunologically active and play an important role in the progression of inflammation and fibrosis. The mRNA of the exosomes reflects the mRNA of the originating cell, so we speculate that the functional difference in Pla-Exos contents and functions between HC and TAO may be due to the changes in circulating immune cell profiles.

We identify miR-144-3p as an immune cell-derived molecule in Pla-Exos that induces Thy-1^+^ OFs dysfunction. Although exosomes contain various proteins and lipids, they are highly enriched in noncoding mRNAs, particularly miRNAs [11, 43]. Exosomes exert their biological actions mainly through their contained miRNAs. Our data highlights that a total of 107 DE-miRNAs that were detected between TAO-A and HC groups. KEGG analysis showed that the target genes of the DE-miRs are mainly enriched in immune response and cell adhesion, which is consistent with the functions of the Pla-Exos. In line with previous reports, our data showed that the main pathological changes in the active phase of TAO are abnormal immune responses [26, 32, 44–46]. RT-qPCR results confirmed that the expression levels of the four exosome-miRNAs (miR-134-5p, miR-144-3p, miR-431-5p and miR-483-3p) were upregulated in patients with active TAO compared to HCs. Their expression levels were further determined in PBMCs to select functional miRNAs derived from immune cells, and the results showed that miR-144-3p was upregulated in TAO-A-PBMCs. Based on these results, we speculate that the upregulated miR-144-3p in Pla-Exos include those of immune cell origin. Surprisingly, we observed no overlap in the specific miRNAs in PBMCs identified by Liu et al., who did not find changes in miR-144 [47]. Among the identified miRNAs, miR-144-3p was most notable as it has been thoroughly investigated and associated with inflammatory response and fibrosis [27, 28, 48, 49]. Thus, miR-144-3p was a candidate mediator of the Thy-1^+^ OFs dysfunction. By means of a mimic-based approach, we provided evidence to miR-144-3p inducing Thy-1^+^ OFs dysfunction; specifically, we demonstrated that miR-144-3p exert proinflammatory and fibrogenic effects and inhibit cell viability of Thy-1^+^ OFs. Our findings are in tandem with those of Chen et al. [27], who demonstrated that upregulation of serum exosomal miR-144-3p as a reliable biomarker of mucosal inflammation in Crohn’s disease (CD), abolished cell viability of human umbilical vein endothelial cells [48]. An emerging study has provided clues that miR-144-3p also enhances cytokine expression in human synovial fibroblasts [49]. Previous studies have shown that miR-144-3p can inhibit cell proliferation [48, 50]. To the best of our knowledge, the present study is the first to identify a novel miRNA enriched in Pla-Exos, providing a basis for the therapeutic application of the plasma exosomes in active TAO.

One limitation of our study was the relatively low sample number, only 25 samples were enrolled for validation, and most of these cases were male (80%), however, the incidence of TAO is significantly lower in males than in females, which may be an additional drawback. Therefore, a large population of active TAO patients will be needed to obtain conclusive evidence of our findings in the future. In addition, in vivo studies have been lacking but are necessary, given the interplay between the different components of the circulatory system and its interaction with different organs in the body. Measuring functional changes in vivo, such as these, is complicated by limited blood volume from patients and the ethical issues associated with rapid, repeated sampling in patients. Critically, further studies will be required to identify the molecular mechanisms underlying the inflammatory and fibrotic effects of Pla-Exos; and the protein cargo will require further analysis because the RNA cargos analysis does not fully explain the function of Pla-Exos function, while proteins may provide more clues to the mechanisms of Pla-Exos action.

## Conclusion

In summary, we identified that active TAO-derived plasma exosomes are immune-active for their structural modifications. The structural modifications (such as up-regulation of miR-144-3p) of exosomes are conceivably causal for the long-term inflammatory and fibrosis progression in patients with TAO. Evidence has revealed that the orbital inflammation and fibrosis process is not unidirectional and permanent in TAO. Currently, there are no “gold standard” therapies for orbital tissue fibrosis, and intravenous corticosteroids are considered the first-line therapy as highlighted by inflammation resolution which can ameliorate orbital fibrosis. In this context, enhancing the clearance of immune-active plasma exosomes or inhibiting the bioactive molecules in plasma exosomes may represent an effective approach for managing active TAO.

### Electronic supplementary material

Below is the link to the electronic supplementary material.

Supplementary Material 1

Supplementary Material 2

Supplementary Material 3

Supplementary Material 4

## Data Availability

The raw sequence data have been deposited in the Genome Sequence Archive in National Genomics Data Center, China National Center for Bioinformation / Beijing Institute of Genomics, Chinese Academy of Sciences (GSA-Human: HRA006037) that are publicly accessible in Gene Expression Omnibus (GEO). Any additional information required to reanalyze the data reported in this paper is available from the corresponding author.
