# Supplementary material for: Plasma exosomes from patients with active thyroid-associated orbitopathy induce inflammation and fibrosis in orbital fibroblasts
Source: J Transl Med. 2024 Jun 7;22:546. doi: 10.1186/s12967-024-05263-y (PMC11157872; doi:10.1186/s12967-024-05263-y)
Supplement: Supplementary file 1 — Supplementary Material 1 [file 12967_2024_5263_MOESM1_ESM.docx]

**The key reagents list**

| **Reagents name** | **Supplier** | **Cat. no.** |
| --- | --- | --- |
| Anti-CD63 antibody | Abcam | ab68418 |
| Anti-TSG101 antibody | Abcam | ab125011 |
| Anti-Alix antibody | Proteintech | 12422-1-AP |
| Anti-Calnexin antibody | Proteintech | 10427-2-AP |
| Horseradish peroxidase-conjugated goat anti-rabbit immunoglobulin G (IgG) | Beyotime | A0208 |
| APC Mouse Anti-Human CD90 (Thy-1) antibody | BD Biosciences | 561971 |
| [APC Mouse IgG1 κ Isotype Control](https://www.bdbiosciences.com/zh-cn/products/reagents/flow-cytometry-reagents/research-reagents/flow-cytometry-controls-and-lysates/apc-mouse-igg1-isotype-control.550854) antibody | BD Biosciences | 550854 |
| Anti-Fibroblast Surface Protein antibody | Sigma-Aldrich | SAB4200821 |
| Anti-Vimentin antibody | Abcam | ab137321 |
| Goat Anti-Mouse IgG H&L / AF488  antibody | Bioss | bs-0296G-AF488 |
| Goat Anti-Rabbit IgG H&L / AF594  antibody | Bioss | bs-0295G-AF594 |
| DAPI Staining Solution | Beyotime | C1006 |
| Phosphate-buffered saline (PBS) | [Biological Industries](http://www.labbase.net/Brand/BrandMode-2110.html) | 02-024-1A |
| Ficoll-Paque^TM^ PREMIUM separation solution | Cytiva | 17544203 |
| Red blood cell lysis buffer | Beyotime | C3702 |
| Dimethylsulfoxide | Sigma-Aldrich | [D8418](https://www.sigmaaldrich.cn/CN/zh/product/sigma/d8418) |
| Pierce BCA Protein Assay Kit | Thermo Fisher Scientific | 23227 |
| RIPA Lysis Assay | Beyotime | P0013B |
| SuperSignal™ West Pico PLUS Chemiluminescent Substrate | Thermo Fisher Scientific | 34580 |
| Dulbecco’s Modified Eagle Medium/Nutrient Mixture F12 (DMEM/F-12) | [Biological Industries](http://www.labbase.net/Brand/BrandMode-2110.html) | 01-170-1A |
| Fetal bovine serum (FBS) | HyClone | SH30396.03 |
| Exosome-depleted FBS | System Biosciences | EXO-FBS-50A-1 |
| GlutaMAX | Gibco | 35050061 |
| Streptomycin & Penicillin | Gibco | 15140122 |
| Trypsin-EDTA | Gibco | 25200056 |
| CD90 MicroBeads | Miltenyi Biotec | 130-096-253 |
| Stain buffer | BD Biosciences | 554656 |
| Antifade Mounting Medium | Beyotime | P0126 |
| PKH67 Green Fluorescent Cell Linker Kit | Sigma-Aldrich | PKH67GL |
| Attractene transfection reagent | QIAGEN | 301005 |
| TRIzol reagent | Beyotime | R0016 |
| HiScript®III RT SuprtMix for qPCR with gDNA wiper | Vazyme | R323-01 |
| Taq Pro Universal SYBR qPCR Master Mix | Vazyme | Q712-02 |
| RNeasy Mini Kit | QIAGEN | 74104 |
| QIAseq miRNA Library Kit | QIAGEN | 331502 |
| SMARTer Stranded Total RNA-Seq Kit v2 | TAKARA | 634414 |
| Human IL-1 beta Quantikine ELISA Kit | R&D Systems | DLB50 |
| Human IL-6 Quantikine ELISA Kit | R&D Systems | D6050 |
| Human TNF-α Quantikine ELISA Kit | R&D Systems | MTA00B-1 |
| Human CXCL2 Quantikine ELISA Kit | R&D Systems | DSA00 |
| Human RANTES Quantikine ELISA Kit | R&D Systems | DRN00B |
| Hyaluronan Quantikine ELISA Kit | R&D Systems | DHYAL0 |
| CCK-8 solution | Beyotime | C0038 |
