# Supplementary material for: Plasma exosomes from patients with active thyroid-associated orbitopathy induce inflammation and fibrosis in orbital fibroblasts
Source: J Transl Med. 2024 Jun 7;22:546. doi: 10.1186/s12967-024-05263-y (PMC11157872; doi:10.1186/s12967-024-05263-y)
Supplement: Supplementary file 2 — Supplementary Material 2 [file 12967_2024_5263_MOESM2_ESM.docx]

**Table S1. Mimics/inhibitors sequences list**

|  | **Sequence** |
| --- | --- |
| miR-144-3p mimic | 5ˈ-UACAGUAUAGAUGAUGUACU-3ˈ |
| NC mimic | 5ˈ-UUUGUACUACACAAAAGUACUG-3ˈ  3ˈ-AAACAUGAUGUGUUUUCAUGAC-5ˈ |
| miR-144-3p inhibitor | 5ˈ-AGUACAUCAUCUAUACUGUA-3ˈ |
| NC inhibitor | 5ˈ-CAGUACUUUUGUGUAGUACAAA-3ˈ |

**Table S2. mRNA primer sequences list**

|  | **Sequence** |
| --- | --- |
| IL-1β | For. GCCAGTGAAATGATGGCTTATT  Rev. AGGAGCACTTCATCTGTTTAGG |
| IL-6 | For. ACTCACCTCTTCAGAACGAATTG  Rev. CCATCTTTGGAAGGTTCAGGTTG |
| TNF-ɑ | For. CCAGGCAGTCAGATCATCTTCTC  Rev. AGCTGGTTATCTCTCAGCTCCAC |
| CXCL1 | For. TTCACAGTGTGTGGTCAACAT  Rev. AAGCCCCTTTGTTCTAAGCCA |
| CXCL2 | For. AGTGTGTGGTCAACATTTCTCA  Rev. GCTCTAACACAGAGGGAAACAC |
| RANTES | For. GCAAGCTTTGTCACCCGAAA  Rev. CCCAAGCTAGGACAAGAGCA |
| CCL8 | For. ATGCTGAAGCTCACACCCTTGCCC  Rev. CAGATGCTTCATGGAATCCCTGACC |
| HAS1 | For. GCGGGCTTGTCAGAGCTAC  Rev. ACTGCTGCAAGAGGTTATTCC |
| HAS3 | For. TTATACAGCTTTTCTACCGGGG  Rev. CAGAAGGCTGGACATATAGAGG |
| ɑ-SMA | For. GGGACTAAGACGGGAATCCT  Rev. TGTCCCATTCCCACCATCAC |
| COL1A1 | For. AAAGATGGACTCAACGGTCTC  Rev. CATCGTGAGCCTTCTCTTGAG |
| GADPH | For. TTGCCATCAATGACCCCTT  Rev. CGCCCCACTTGATTTTGGA |

For., forward; Rev., reverse.

**Table S3. miRNA primer sequences List**

| **Name** | **Sequence** |
| --- | --- |
| hsa-miR-34α-5p | RT: CTCAACTGGTGTCGTGGAGTCGGCAATTCAGTTGAGACAACCAG |
|  | For. ACACTCCAGCTGGGTGGCAGTGTCTTAGCT |
| hsa-miR-218-5p | RT: CTCAACTGGTGTCGTGGAGTCGGCAATTCAGTTGAGACATGGTT |
|  | For. ACACTCCAGCTGGGTTGTGCTTGATCTAA |
| hsa-miR-7α-3p | RT: CTCAACTGGTGTCGTGGAGTCGGCAATTCAGTTGAGGAAAGACA |
|  | For. ACACTCCAGCTGGGCTATACAATCTACTG |
| hsa-miR-483-3p | RT: CTCAACTGGTGTCGTGGAGTCGGCAATTCAGTTGAGAAGACGGG |
|  | For. ACACTCCAGCTGGGTCACTCCTCTCCTCC |
| hsa-miR-144-3p | RT: CTCAACTGGTGTCGTGGAGTCGGCAATTCAGTTGAGAGTACATC |
|  | For. ACACTCCAGCTGGGTACAGTATAGATGA |
| hsa-miR-134-5p | RT: CTCAACTGGTGTCGTGGAGTCGGCAATTCAGTTGAGCCCCTCTG |
|  | For. ACACTCCAGCTGGGTGTGACTGGTTGACCA |
| hsa-miR-432-5p | RT: CTCAACTGGTGTCGTGGAGTCGGCAATTCAGTTGAGCCACCCAA |
|  | For. ACACTCCAGCTGGGTCTTGGAGTAGGTCATT |
| hsa-miR-25-5p | RT: CTCAACTGGTGTCGTGGAGTCGGCAATTCAGTTGAGCAATTGCC |
|  | For. ACACTCCAGCTGGGAGGCGGAGACTTGGG |
| hsa-miR-382-5p | RT: CTCAACTGGTGTCGTGGAGTCGGCAATTCAGTTGAGCGAATCCA |
|  | For. ACACTCCAGCTGGGGAAGTTGTTCGTGGTG |
| hsa-miR-485-3p | RT: CTCAACTGGTGTCGTGGAGTCGGCAATTCAGTTGAGAGAGAGGA |
|  | For. ACACTCCAGCTGGGGTCATACACGGCTCTC |
| hsa-miR-6852-5p | RT: CTCAACTGGTGTCGTGGAGTCGGCAATTCAGTTGAGCATGTCCT |
|  | For. ACACTCCAGCTGGGCCCTGGGGTTCTGAG |
| hsa-miR-504-5p | RT: CTCAACTGGTGTCGTGGAGTCGGCAATTCAGTTGAGGATAGAGT |
|  | For. ACACTCCAGCTGGGAGACCCTGGTCTGCAC |
| hsa-miR-431-5p | RT: CTCAACTGGTGTCGTGGAGTCGGCAATTCAGTTGAGTGCATGAC |
|  | For. ACACTCCAGCTGGGTGTCTTGCAGGCCGT |
| hsa-miR-370-3p | RT: CTCAACTGGTGTCGTGGAGTCGGCAATTCAGTTGAGACCAGGTT |
|  | For. ACACTCCAGCTGGGGCCTGCTGGGGTGGAA |
| hsa-miR-625-3p | RT: CTCAACTGGTGTCGTGGAGTCGGCAATTCAGTTGAGTGAGGGGG |
|  | For. ACACTCCAGCTGGGGACTATAGAACTTTCC |
| hsa-miR-33a-5p | RT: CTCAACTGGTGTCGTGGAGTCGGCAATTCAGTTGAGTGCAATGC |
|  | For. ACACTCCAGCTGGGGTGCATTGTAGTTGC |
| U6 | RT: CTCAACTGGTGTCGTGGAGTCGGCAATTCAGTTGAGAAAATATG |
|  | For. CAAATTCGTGAAGCGTT |
| miRNA-URP1 | TGGTGTCGTGGAGTCG |

**Table S4. List of databases used for miRNA research**

| **Name** | **Link** |
| --- | --- |
| Human Genome | http://genome.ucsc.edu |
| Silva | https://www.arb-silva.de/ |
| GtRNAdb | http://gtrnadb.ucsc.edu |
| Rfam | https://rfam.org/ |
| Repbase | https://www.girinst.org/repbase/ |
| Human Genome | http://genome.ucsc.edu |
| miRBase | https://www.mirbase.org/ |
| Targetscan | http://www.targetscan.org/ |
| miRanda | http://www.microrna.org/microrna/home.do/ |
| PicTar | http://pictar.mdc-berlin.de/ |
| MicroCosm Targets | http://www.ebi.ac.uk/enright-srv/microcosm/htdocs/targets/v5/ |
| miRDB | http://mirdb.org/ |
| KEGG | http://www.genome.jp/kegg |
| miRcode | http://www.mircode.org/ |

**Table S5. Patient populations studied**

|  | HCs | Active TAO |
| --- | --- | --- |
|  | | |
| Functional study, RNA content discovery study | | |
| N | 5 | 5 |
| Female / Male | 1/4 | 1/4 |
| Age (mean ± SD) | 51.00 ± 9.97 | 51.00 ± 11.60 |
| CAS, median (range)^b^ | NA | 4.4 (4 – 5) |
| Disease Severity | NA | moderate-to-severe |
| Disease duration, mean (SD) [range] | NA | 1.54 (0.2 – 2) |
|  | | |
| Exosome-miRNA validation Study | | |
| N | 20 | 20 |
| Female / Male | 7/13 | 7/13 |
| Age (mean ± SD), year | 47.55 ± 7.61 | 47.75 ± 7.85 |
| CAS, median (range) | NA | 4.2 (3 – 5) |
| Disease Severity | NA | moderate-to-severe |
| Disease duration, mean (SD) [range] | NA | 1.24 (0.5 – 2.5) |
|  | | |
| PBMCs-miRNA validation study | | |
| N | 10 | 10 |
| Female / Male | 3/7 | 3/7 |
| Age (mean ± SD) | 47.00 ± 7.92 | 49.00 ± 8.23 |
| CAS, median (range)^a^ | NA | 3.9 (3 – 5) |
| Disease Severity | NA | moderate-to-severe |
| Disease duration, mean (SD) [range] | NA | 1.06 (0.2 – 2) |

TAO, thyroid-associated ophthalmopathy; SD, Standard deviation; NA, not applicable; CAS, clinical activity score; PBMCs, peripheral blood mono-nuclear cells. CAS, clinical activity score

^a^ Range, 0 to 7; TAO active for clinical activity score values of 3 or higher

**Table S6. Differently expressed immune-related maker genes list in patients with active TAO vs HC**

| **Symbol** | **Fold change** | ***p* value** | **FDR** |
| --- | --- | --- | --- |
| HLA-DMB | -4.5^†^ | 0.010 | 0.020 |
| TREM1 | -3.2 | 0.039 | 0.040 |
| CD300E | -2.7 | 0.016 | 0.026 |
| BST2 | -2.4 | 0.036 | 0.040 |
| IGFBP5 | -2.1 | 0.037 | 0.040 |
| HLA-DPA1 | -1.9 | 0.024 | 0.032 |
| RALB | -1.7 | 0.029 | 0.038 |
| FERMT3 | 2.4 | 0.040 | 0.040 |
| NCOA4 | 2.4 | 0.020 | 0.028 |
| ARHGAP10 | 2.7 | 0.040 | 0.040 |
| DYNLL1 | 2.9 | 0.006 | 0.016 |
| DAB2 | 3.0 | 0.019 | 0.028 |
| CA2 | 3.3 | 0.015 | 0.026 |
| PTGS1 | 3.5 | 0.008 | 0.018 |
| MMD | 3.6 | 0.010 | 0.020 |
| NAA16 | 3.6 | 0.031 | 0.039 |
| CCL5 | 3.9 | 0.002 | 0.008 |
| FSTL1 | 4.0 | 0.014 | 0.026 |
| F13A1 | 4.0 | 0.005 | 0.015 |
| DAPP1 | 4.4 | 0.003 | 0.011 |
| ITGB3 | 4.5 | 0.003 | 0.019 |
| ITGB1 | 4.5 | 0.002 | 0.008 |
| MFAP3L | 4.7 | 0.005 | 0.015 |
| RAB27B | 5.1 | 0.002 | 0.008 |
| ITGA2B | 5.4 | 0.002 | 0.008 |
| SFXN3 | 60.8 | 0.000 | 0.000 |
| AHCYL2 | 130.0 | 0.000 | 0.000 |

^†^ “-” indicates that exosomal miRNA expression is down-regulated in the TAO-A group compared with the HC group

**Table S7. Immune cells related gene makers list**

| **Cell types** | **Maker genes** |
| --- | --- |
| Neutrophil | CREB5, CDA, CHST15, S100A12, APOBEC3A, CASP5, MMP25, HAL, C1orf183, FFAR2, MAK, CXCR1, STEAP4, MGAM, BTNL8, CXCR2, TNFRSF10C, VNN3 |
| Activated  CD4^+^ T cell | AIM2, BIRC3, BRIP1, CCL20, CCL4, CCL5, CCNB1, CCR7, DUSP2, ESCO2, ETS1, EXO1, EXOC6, IARS, ITK, KIF11, KNTC1, NUF2, PRC1, PSAT1, RGS1, RTKN2, SAMSN1, SELL, TRAT1 |
| Th1 | CD70, TBX21, ADAM8, AHCYL2, ALCAM, B3GALNT1, BBS12, BST1, CD151, CD47, CD48, CD52, CD53, CD59, CD6, CD68, CD7, CD96, CFHR3, CHRM3, CLEC7A, COL23A1, COL4A4, COL5A3, DAB1, DLEU7, DOC2B, EMP1, F12, FURIN, GAB3, GATM, GFPT2, GPR25, GREM2, HAVCR1, HSD11B1, HUNK, IGF2, RCSD1, RYR1, SAV1, SELE, SELP, SH3KBP1, SIT1, SLC35B3, SIGLEC10, SKAP1, THUMPD2, TIGIT, ZEB2, ENC1, FAM134B, FBXO30, FCGR2C, STAC, LTC4S, MAN1B1, MDH1, MMD, RGS16, IL12A, P2RX5, CD97, ITGB4, ICAM3, METRNL, TNFRSF1A, IRF1, HTR2B, CALD1, MOCOS, TRAF3IP2, TLR8, TRAF1, DUSP14 |
| Th2 | ASB2, CSRP2, DAPK1, DLC1, DNAJC12, DUSP6, GNAI1, LAMP3, NRP2, OSBPL1A, PDE4B, PHLDA1, PLA2G4A, RAB27B, RBMS3, RNF125, TMPRSS3, GATA3, BIRC5, CDC25C, CDC7, CENPF, CXCR6, DHFR, EVI5, GSTA4, HELLS, IL26, LAIR2 |
| Th17 | IL17A, IL17RA, C2CD4A, C2CD4B, CA2, CCDC65, CEACAM3, IL17C, IL17F, IL17RC, IL17RE, IL23A, ILDR1, LONRF3, SH2D6, TNIP2, ABCA1, ABCB1, ADAMTS12, ANK1, ANKRD22 , B3GALT2, CAMTA1, CCR9, CD40, GPR44 , IFT80 |
| Treg | CCL3L1, CD72, CLEC5A, FOXP3, ITGA4, L1CAM, LIPA, LRP1, LRRC42, MARCO, MMP12, MNDA, MRC1, MS4A6A, PELO, PLEK, PRSS23, PTGIR, ST8SIA4, STAB1 |
| Tfh | B3GAT1, CDK5R1, PDCD1, BCL6, CD200, CD83, CD84, FGF2, GPR18, CEBPA, CECR1, CLEC10A, CLEC4A, CSF1R, CTSS, DMN, DPP4, LRRC32, MC5R, MICA, NCAM1, NCR2, NRP1, PDCD1LG2, PDCD6, PRDX1, RAE1, RAET1E, SIGLEC7, SIGLEC9, TYRO3, CHST12, CLIC3, IVNS1ABP, KIR2DL2, LGMN |
| CD4^+^ T_CM_ | ABHD3, AHNAK, ANXA2P2, AQP3, ATHL1, BMI1, BZW2, CD63, COL4A1, CYLD, ELMO2, FYN, GLIPR1, GSS, IFITM2, ITGB1, ITGB2, KLF5, LSP1, NDUFB9, PKM2, SFXN3, SIRPG, SMAD4, STX4, TRADD, VIM, XRCC6 |
| CD4^+^ T_EM_ | ATM, CASP3, CASQ1, CD300E, DARS, DOCK9, EXOSC9, EZH2, GDE1, IL34, NCOA4, NEFL, PDGFRL, PTGS1, REPS1, SCG2, SDPR, SIGLEC14, SIGLEC6, TAL1, TFEC, TIPIN, TPK1, UQCRB, USP9Y, WIPF1, ZCRB1 |
| Activated  CD8^+^ T cell | ADRM1, AHSA1, C1GALT1C1, CCT6B, CD37, CD3D, CD3E, CD3G, CD69, CD8A, CETN3, CSE1L, GEMIN6, GNLY, GPT2, GZMA, GZMH, GZMK, IL2RB, LCK, MPZL1, NKG7, PIK3IP1, PTRH2, TIMM13, ZAP70 |
| CD8^+^ T_CM_ | ACTN4, ADAM12, ADCY9, F13A1, FCER1G, FCGR3B, FGF7, FKBP4, GLUD1, GM2A, GUSB, IL1RN, NOL11, NTRK1, RARA, RNF128, SIGLEC1, TNFRSF11A, TOX4, UBA52, ULBP1 |
| CD8^+^ T_EM_ | ACAP1, APOL3, ARHGAP10, ATP10D, C3AR1, CCR5, CD160, CD55, CFLAR, CMKLR1, DAPP1, FCRL6, FLT3LG, GZMM, HAPLN3, HLA-DMB, HLA-DPA1, HLA-DPB1, IFI16, LIME1, LTK, NFKBIA, SETD7, SIK1, TRIB2 |
| γδ T cell | ACP5, AQP9, BTN3A2, C1orf54, CARD8, CCL18, CD209, CD33, CD36, CDK5, IL10RB, KLRF1, LGALS1, MAPK7, KLHL7, KRT80, LAMC1, LCORL, LMNB1, MEIS3P1, MPL, FABP1, FABP5, FADD, MFAP3L, MINPP1, RPS24, RPS7, RPS9, DBNL, CCL13 |
| Immature B cell | CD22, CYBB, FAM129C, FCRL1, FCRL3, FCRL5, FCRLA, HDAC9, HLA-DQA1, HVCN1, KIAA0226, NCF1, NCF1B, P2RY10, SP100, TXNIP, STAP1, TAGAP, ZCCHC2 |
| Activated B cell | ADAM28, CD180, CD79B, BLK, CD19, MS4A1, TNFRSF17, IGHM, GNG7, MICAL3, SPIB, HLA-DOB, IGKC, PNOC, FCRL2, BACH2, CR2, TCL1A, AKNA, ARHGAP25, CCL21, CD27, CD38, CLEC17A, CLEC9A, CLECL1 |
| Memory B cell | AICDA, CCNA2, CDKN3, CLCN5, ENPP1, FCER1A, FCRL4 , MYC, RUNX2, SORL1, SOX5, STAT5A, STAT5B, TLR9 |
| Mo | ASGR2, CFP, ASGR1, CD1D, UPK3A, ACTG1, ANXA5, ATP6V1B2, CFL1, DAZAP2, CTBS, EMR4P, HIVEP2, MARCKSL1, MBP, MMP15, PNPLA6, TMBIM6, PQBP1, TEX264, IKZF1 |
| Macrophage | AIF1, CCL1, CCL14, CCL23, CCL26, CD300LB, CNR1, CNR2, EIF1, EIF4A1, FPR1, FPR2, FRAT2, GPR27, GPR77, RNASE2, MS4A2, BASP1, IGSF6, HK3, VNN1, FES, NPL, FZD2, FAM198B, HNMT, SLC15A3, CD4, TXNDC3, FRMD4A, CRYBB1, HRH1, WNT5B |
| Immature  DC | ACADM, AHCYL1, ALDH1A2, ALDH3A2, ALDH9A1, ALOX15, AMT, ARL1, ATIC, ATP5A1, CAPZA1, LILRA5, RDX, RRAGD, TACSTD2, INPP5F, RAB38, PLAU, CSF3R, SLC18A2, AMPD2, CLTB, C1orf162 |
| Activated DC | ABCD1, C1QC, CAPG, CCL3L3, CD207, CD302, ATP5B, ATP5L, ATP6V1A, BCL2L1, C1QB, SNURF, SPCS3, CCNA1, CEACAM8 , NOS2, SRA1, TNFRSF6B, TREM1, TREML1, RHOA, SLC25A37, TNFSF14, TREML4, VNN2, XPO6, CLEC4C, TNFAIP2, UBD, ACTR3, RAB1A, SLA, HLA-DQA2, SIGLEC5, SLAMF9 |
| pDC | CBX6, DAB2, DDX17, HIGD1A, IDH3A, IL3RA, MAGED1, NUCB2, OFD1, OGT, PDIA4, SERTAD2, SIRPA, TMED2, ENG, FCAR, IGF1, ITGA2B, GABARAP, GPX1, KRT23, PROK2, RALB, RETNLB, RNF141, SEC14L1, SEPX1, EMP3, CD300LF, ABTB1, KLHL21, PHRF1 |
| NK cell | AKT3, AXL, BST2, CDH2, CRTAM, CSF2RA, CTSZ, CXCL1, CYTH1, DAXX, DGKH, DLL4, DPYD, ERBB3 , F11R, FAM27A, FAM49A, FASLG, FCGR1A, FN1, FSTL1, FUCA1, GBP3, GLS2, GRB2, LST1, BCL2, CDC5L, FGF18, FUT5, FZR1, GAGE2, IGFBP5, KANK2, LDB3 |
| CD56^+^  NK cell | ABAT, C11orf75, C5orf15, CDHR1, DCAF12, DYNLL1, GPR137B, HCP5, HDGFRP2, KRT86, MLST8, ELMOD3, ENTPD5, FAM119A, FAM179A, CLIC2, COX7A2L, CREB3L4, CSF1 , CSNK2A2, CSTA, CSTB, CTPS, CTSD, FST, GATA2, GMPR, HDC, HEY1, HOXA1, HS2ST1, HS3ST1, BCL11B, CDH3, MYL6B, NAA16, ClQA, ClQB, CYP27B1, EIF3M |
| CD56^–^  NK cell | CYP27A1, DDX55, DYRK2, RPL37A, NOTCH3, AKR7A3, GPRC5C, GRIN1, HLA-E, PORCN, PSMC4, UPP1, IL21R, KIR2DS1, KIR2DS2, KIR2DS5 |
| NKT cell | BTN2A2, CD101, CD109, CNPY3, CNPY4, CREB1, CRTC2, CRTC3, CSF2, KLRC1, FUT4, ICAM2, IL32, LAMP2, LILRB5, KLRG1, HSPA4, HSPB6, ISM2, ITIH2, KDM4C, KIR2DS4, KIRREL3, SDCBP, NFATC2IP, MICB, KIR2DL1, KIR2DL3, KIR3DL1, KIR3DL2, NCR1, FOSL1, TSLP, SLC7A7, SPP1, TREM2, UBASH3A, YBX2, CCDC88A, CLEC1A, THBD, PDPN, VCAM1, EMR1 |
| Mast cell | ADAMTS3, CPA3, CMA1, CTSG, ARHGAP15, CPM, FCN1, FTL, HSPA6, ITGA9, RNASE3, S100A4, SIGLEC8, SLC6A4, PTGS2, EGR3, PILRA |
| MDSC | CCR2, CD14, CD2, CD86, CXCR4, FCGR2A, FCGR2B, FCGR3A, FERMT3, GPSM3, IL18BP, IL4R, ITGAL, ITGAM, PARVG, PSAP, PTGER2, PTGES2, S100A8, S100A9 |
| Esoinophil | GIPR , KRT18P50, LRMP, FOSB, RRP12, GPR183, NR4A3, ST3GAL6, DEPDC5, PDE6C, PKD2L2, GPR65, IL5RA, P2RY14, DACH1, DAPK2, EMR3 |
| Endothelial cell | PECAM1, VMF, ACKR1, CLDN5, TM4SF1, JAG1, RBP7, FABP4, SOCS3, SPRY1, HSPA6, ACP5, INMT, PLCG2, CD34, ITGB3, ICAM1, SELE, ENG, VCAM1, CDH5, MCAM |

Th, T helper cell; Treg, regular T cell; Tfh, T follicular helper cell; T_CM_, central memory T cell; T_EM_, effector memory T cell; Mo, monocyte; DC, dendritic cell; pDC, plasmacytoid dendritic cell; NK, natural killer; NKT, natural killer T; MDSC, myeloid-derived suppressor cell
