# Supplementary material for: Plasma exosomes from patients with active thyroid-associated orbitopathy induce inflammation and fibrosis in orbital fibroblasts
Source: J Transl Med. 2024 Jun 7;22:546. doi: 10.1186/s12967-024-05263-y (PMC11157872; doi:10.1186/s12967-024-05263-y)
Supplement: Supplementary file 3 — Supplementary Material 3 [file 12967_2024_5263_MOESM3_ESM.docx]

**
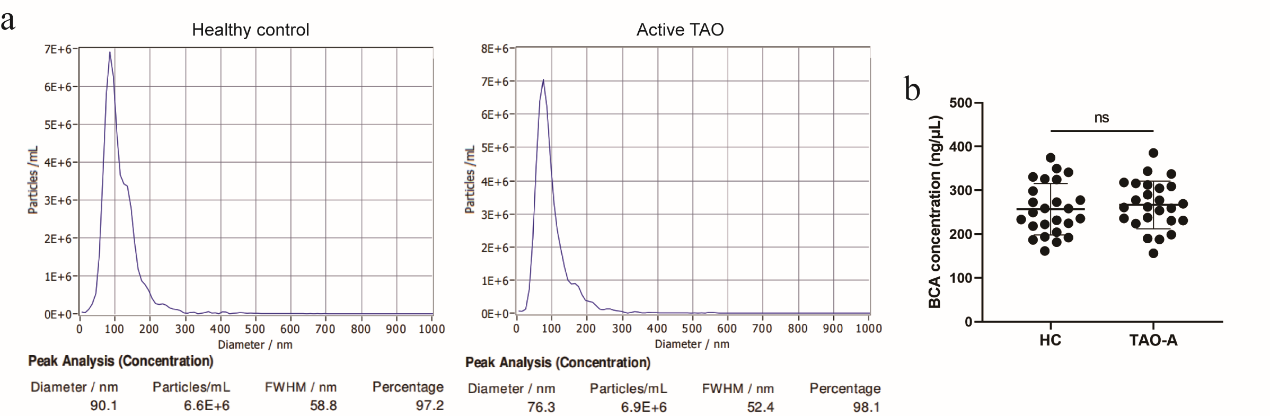
**

**Fig. S1 Concentration analysis of plasma exosomes from patients with active TAO and healthy controls. (a)** Exosome size distribution was determined through nanoparticle tracking analysis. **(b)** Exosome concentrations were determined using a BCA protein assay kit. ns, no significant difference.

**
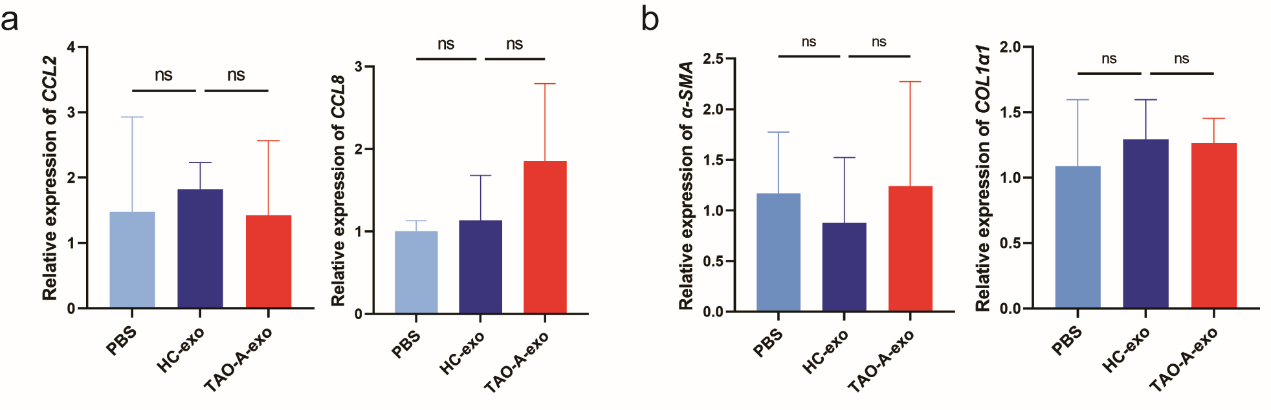
Fig. S2 The function of plasma exosomes on Thy-1^+^ orbital fibroblasts. (a)** Levels of CCL2 and CCL8 in Thy-1^+^ OFs treated with Pla-Exos derived from active TAO patients and HCs. **(b)** Pla-Exos derived from active TAO patients limited the fibrogenic phenotype in Thy-1^+^ OFs. Levels of α-SMA and COL1α1 in Thy-1^+^ OFs treated with Pla-Exos derived from active TAO patients and HCs. The relative expression of the maker genes was determined by RT-PCR and normalized to are normalized to GADPH mRNA levels. OF orbital fibroblasts, Pla-Exos plasma exosomes, HCs healthy controls, α-SMA α-smooth muscle actin, COL1α1 collagen 1α1.

**
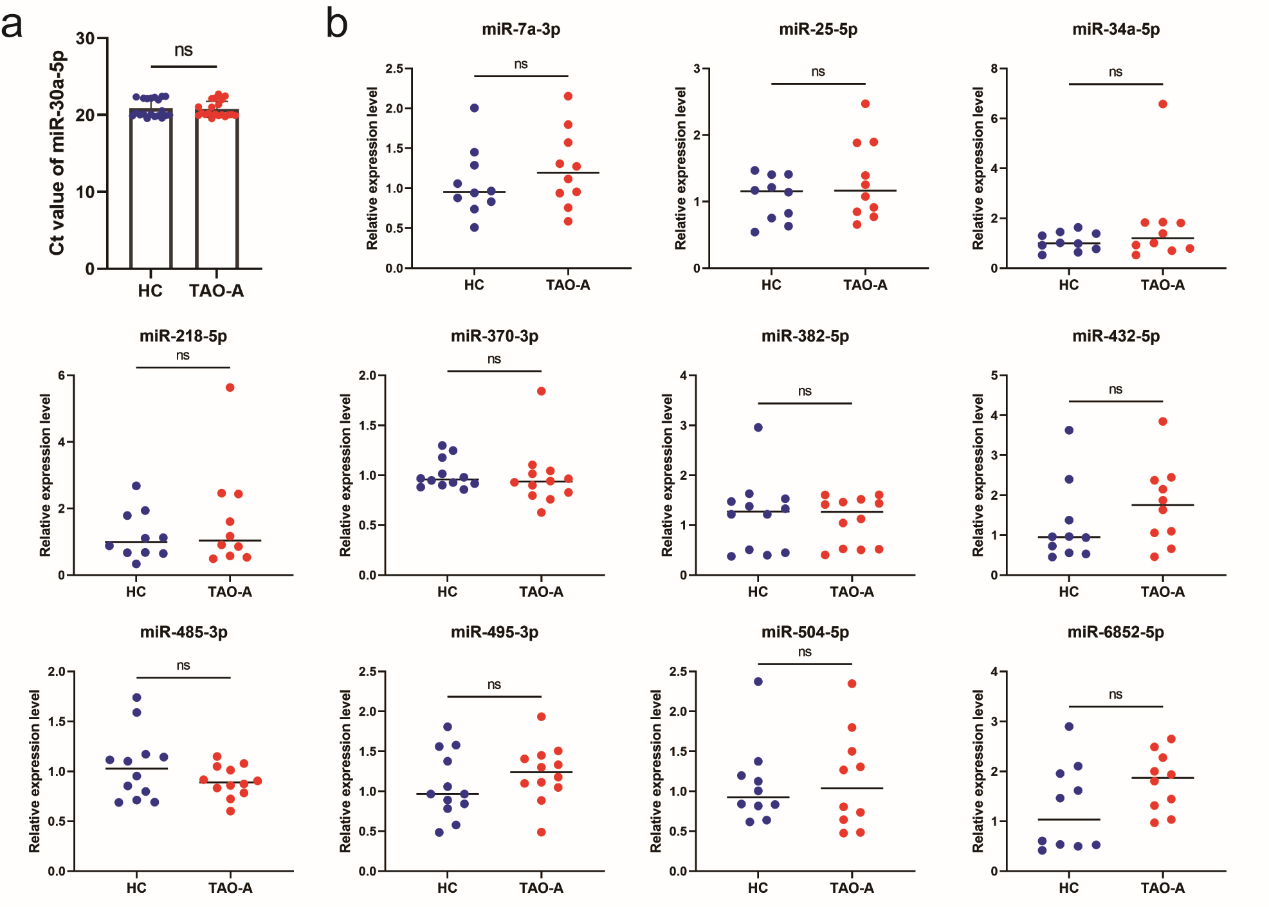
Fig. S3 RT-qPCR analysis for the differently expressed miRNAs. (a)** Ct value of miR-30a-5p in Pla-Exos derived from patients with active TAO and HCs. **(b)** Levels of the Differently expressed miRNA determined by RT-PCR and normalized to miR-30a-5p levels. OF orbital fibroblasts, Pla-Exos plasma exosomes, HCs healthy controls.

**
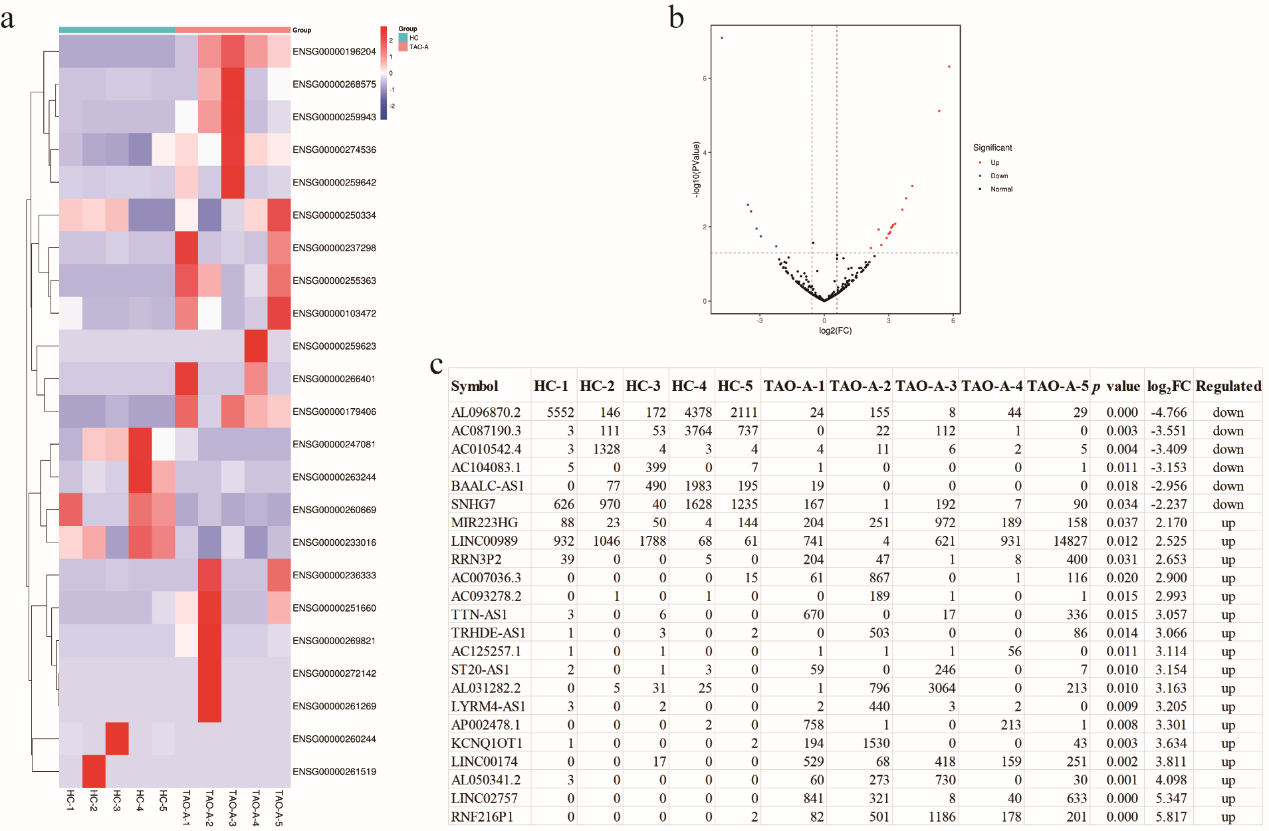
Fig. S4 Differently expressed lncRNAs in plasma exosomes from patients with active TAO and healthy controls. (a)** Heat map of lncRNA expression profile in Pla-Exos derived from plasma of 5 HCs and 5 patients with active TAO. RNA-seq was used for lncRNA content analysis. **(b)** Volcano plot comparing expressed lncRNAs in Pla-Exos from patients with active TAO and HCs. The volcano plot was created using a log2 fold change and –log10 *P* values of all the detected lncRNAs. **(c)** Expression level distribution of the differently expressed lncRNA per sample.

**
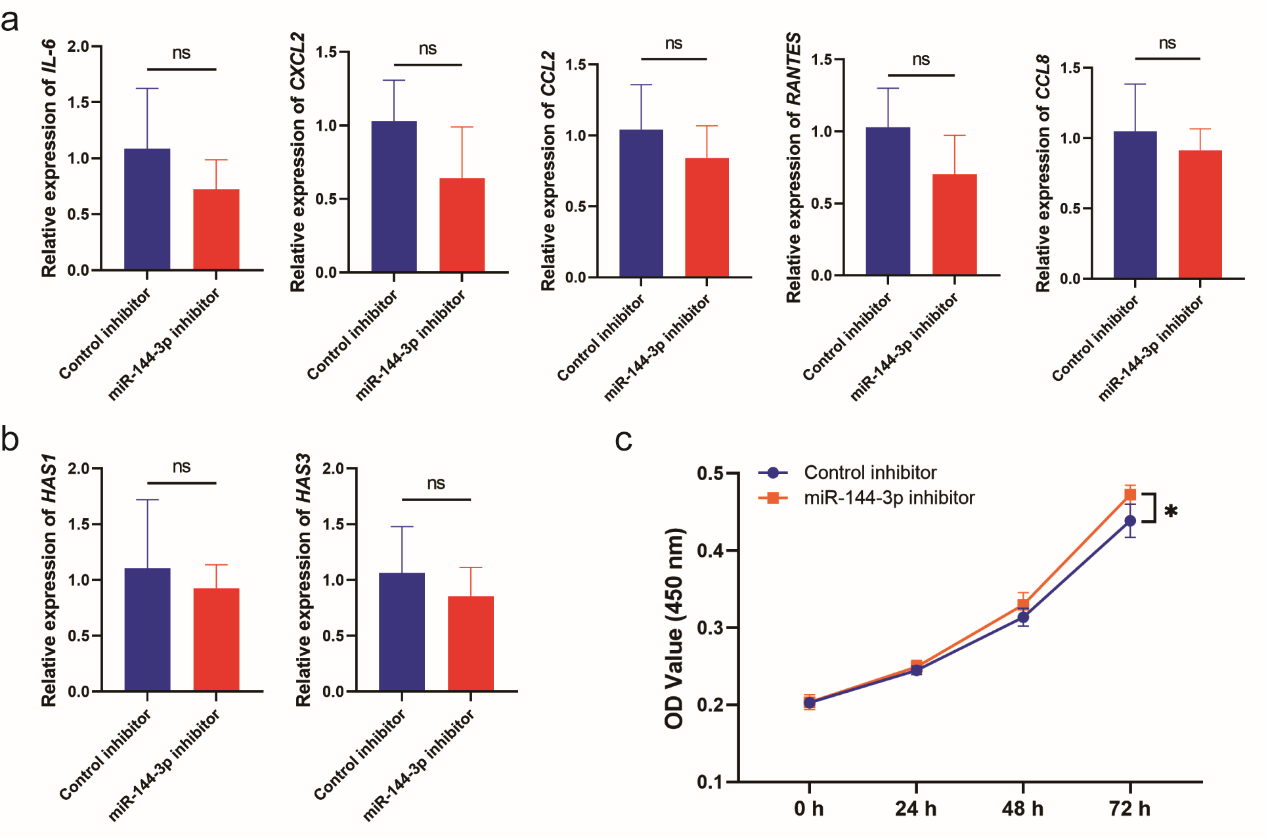
Fig. S5 (a)** Levels of IL-6, CXCL2, CCL2, RANTES and CCL8 in Thy-1+ OFs treated with miR-144-3p inhibitors or control inhibitors. **(b)** Levels of HAS1 and HAS3 in Thy-1^+^ OFs treated with treated with miR-144-3p inhibitors or control inhibitors. The relative expression of the maker genes was determined by RT-PCR and normalized to GADPH mRNA levels. OF orbital fibroblasts. **(c)** Cell proliferation was measured by CCK-8 in Thy-1^+^ OFs treated with miR-144-3p inhibitors or control inhibitors.
