# Supplementary material for: Plasma exosomes from patients with active thyroid-associated orbitopathy induce inflammation and fibrosis in orbital fibroblasts
Source: J Transl Med. 2024 Jun 7;22:546. doi: 10.1186/s12967-024-05263-y (PMC11157872; doi:10.1186/s12967-024-05263-y)
Supplement: Supplementary file 4 — Supplementary Material 4 [file 12967_2024_5263_MOESM4_ESM.pdf]

受试者知情同意书

项目名称：循环外泌体中 ncRNA 和蛋白质作为甲状腺相关性眼病的调控因子和治疗靶点研究

方案版本号及版本日期：版本号：1.0，版本日期：2021.07.10

知情同意书版本号及版本日期：版本号：1.0，版本日期：2021.07.10

尊敬的受试者：

我们将要开展一项关于循环外泌体中 ncRNA 和蛋白质作为甲状腺相关性眼病的调控因子和治疗靶点的研究，您的具体情况符合该项研究的入组条件，因此，我们想邀请您参加该项研究。本知情同意书将向您介绍该研究的目的、步骤、获益、风险、不便或不适等，请仔细阅读后慎重做出是否参加研究的决定。当研究人员向您说明和讨论知情同意书时，您可以随时提问并让他/她向您解释您不明白的地方。您可以与家人、朋友以及您的经治大夫讨论之后再做决定。

1. 为什么进行这项研究？

甲状腺相关性眼病（Thyroid associated ophthalmopathy，TAO）是一种器官特异性自身免疫性疾病。其发病率高，占眼眶疾病的 20%，是成人患病率最高的眼眶疾病。常引起眼睑退缩、眼球突出、眼球运动障碍、压迫性视神经病变等症状和体征，威胁视力，严重影响患者的美观和生活质量。TAO 属眼科难治性疾病之一，目前主要采用糖皮质激素治疗，但是治疗效果不确切，并且容易复发，对于中重度和极重度患者，还需手术治疗来改善视力和外观，会给家庭和社会带来沉重的经济负担。病因治疗是改善 TAO 的关键所在，本项目拟进行了关于循环外泌体中 ncRNA 和蛋白质作为 TAO 的调控因子和治疗靶点的研究，目的是为探索 TAO 患者循环血 Exo 中特征性、功能性 ncRNA 和蛋白质作为 TAO 治疗靶点的潜能。

2. 哪些人将被邀请参加这项研究？

就诊于温州医科大学附属眼视光医院，确诊为 TAO 的患者。纳入标准：①TAO 确诊患者；②既往存在 Graves 病病史；③采血前 3 个月内未接受过糖皮质激素/免疫抑制剂治疗的患者；④40~60 岁。排除标准：①全身或眼眶免疫性/过

敏性疾病、慢性炎症性疾病、肿瘤病史；②近期全身严重感染病史。

3. 多少人将参与这项研究？

本研究计划招募约 150 名受试者。

4. 本研究包括哪些内容？

本研究需从门诊病历和/或住院病历搜集患者的性别、年龄、眼部症状和体征、甲亢病史、治疗史等信息，需采集外周血，以及具备手术指证行手术治疗的患者的眶脂肪组织（为达到治疗目的而去除的部分脂肪组织），所有患者首次就诊时空腹采集外周血 10ml，活动期患者治疗后空腹状态下再次采集外周血 10ml，手术治疗中采集眶内脂肪组织。

5. 这项研究会持续多久？

外周血采集约耗时 5 分钟结束；眶脂肪组织采集于术中进行，手术结束即终止。

6. 参加本项研究的风险是什么？

可能出现局部出血或青紫。尽管可能性很小，也可能出现感染、出血过多、凝血或晕厥的情况。采血过程中准备止血带、消毒用酒精或碘伏、以及紧急抢救的设备，若出现上述情况则立即压迫止血，局部抗炎，必要时给予紧急抢救，青紫可自行消退无需特殊处理。

7. 参加本项研究的获益是什么？

参加本研究将提供受试者健康咨询或相关医疗保健等服务。参与本研究有助于探索 TAO 的发病机制，为 TAO 的特异性靶向治疗和对因治疗提供可能性。

8. 是否一定要参加并完成本项研究？

您是否参加这个研究完全是自愿的。如果您不愿意，可以拒绝参加，这对您目前或未来的医疗不会有任何负面影响。即使您同意参加以后，您也可以在任何时间改变主意，告诉研究者退出研究，您的退出不会影响您获得正常的医疗服务。原则上，在您退出之后，研究者将严密保存您的相关信息直至最终销毁，期间不会继续使用或透露这些信息。但是出现以下极少数情况时，研究者将继续使用或透露您的相关信息，即使您已经退出研究或研究已经结束，这些情况包括：

①除去您的信息将影响研究结果的科学性或对数据安全的评价；

②为研究、教学或其他活动提供一些有限的信息（这些信息不会包括您的姓名、身份证号码、或者其他能识别您身份的个人信息）；

③一旦出现任何可能会影响您决定是否继续参加该项研究的信息，我们会及时告知您。

9. 关于研究费用和补偿

本研究涉及的外周血和眶脂肪组织采集等费用由研究者承担，在患者加入本项受试前，会与其进行详细的说明。无交通费用、误工费用等补偿。

10. 参加该项研究受试者是否获得报酬？

无。

11. 发生研究相关伤害的处理？

当您的健康状况因参加本研究而受到伤害时，请告知研究者涂云海医生，联系电话：18243057380，我们会采取必要的医疗措施。根据我国相关法规条例规定，发生研究相关的伤害时，本项研究的申办方将承担相应的医疗费用及对此提供相应的经济补偿。

12. 我的信息会保密吗？

如果您决定参加本项研究，您参加研究及在研究中的个人资料均属保密。您的血/尿标本将以研究编码而非您的姓名加以标识。在未获得您的许可之前，任何可以识别您身份的信息将不会透露给研究小组以外的成员。所有的研究成员和研究相关方都会按要求对您的身份保密。您的档案将保存在有锁的档案柜中，仅供研究人员查阅。为确保研究按照规定进行，必要时，政府管理部门、学校当局或伦理委员会的成员按规定可以在研究单位查阅您的个人资料。这项研究结果发表时，将不会披露您个人的任何资料。

13. 如果我有问题或困难，该与谁联系？

如果您有与本研究相关的任何问题，请联系涂云海医生，联系电话18243057380。

如果您有与受试者自身权益相关的问题或在研究过程中对研究人员有抱怨，可与温州医科大学附属眼视光伦理委员会联系，联系电话：0577-88075582。

受试者签字页

受试者同意声明：

☑ 我已经阅读了上述有关本研究的介绍，且研究医生已向我详细地讲解了研究内容，在签署知情同意书前我已没有更多有关研究的疑惑需咨询。在此基础上，我自愿参加本文所介绍的临床研究，并且我的决定是基于对参加本研究可能产生的风险和受益充分了解。此外，研究者没有对我使用欺骗、利诱、胁迫等手段强行让我同意参加研究，并且我知道我可以在任何阶段无条件退出研究。

☐ 该名受试者因无行为能力、限制行为能力，本知情同意由其监护人或者法定代理人代为签署。

受试者签名：[illegible]　　法定代理人签名：

日　　期：2022.3.16　　日　　期：

受试者联系方式：13078574298　　法定代理人联系方式：

研究者声明：

我确认已向患者解释了本研究的详细情况，特别是参加本研究可能产生的风险和收益。

研究者签名：[illegible]

日　　期：2022.3.16

研究者联系方式：13784174738

受试者签字页

受试者同意声明：

☑ 我已经阅读了上述有关本研究的介绍，且研究医生已向我详细地讲解了研究内容，在签署知情同意书前我已没有更多有关研究的疑惑需咨询。在此基础上，我自愿参加本文所介绍的临床研究，并且我的决定是基于对参加本研究可能产生的风险和受益充分了解。此外，研究者没有对我使用欺骗、利诱、胁迫等手段强行让我同意参加研究，并且我知道我可以在任何阶段无条件退出研究。

☐ 该名受试者因无行为能力、限制行为能力，本知情同意由其监护人或者法定代理人代为签署。

受试者签名：吴金花　　法定代理人签名：______

日　　期：2022.5.6　　日　　期：______

受试者联系方式：15887859885　　法定代理人联系方式：______

研究者声明：

我确认已向患者解释了本研究的详细情况，特别是参加本研究可能产生的风险和收益。

研究者签名：[illegible]

日　　期：2022.5.6

研究者联系方式：13780174738

受试者签字页

受试者同意声明：

☑ 我已经阅读了上述有关本研究的介绍，且研究医生已向我详细地讲解了研究内容，在签署知情同意书前我已没有更多有关研究的疑惑需咨询。在此基础上，我自愿参加本文所介绍的临床研究，并且我的决定是基于对参加本研究可能产生的风险和受益充分了解。此外，研究者没有对我使用欺骗、利诱、胁迫等手段强行让我同意参加研究，并且我知道我可以在任何阶段无条件退出研究。

☐ 该名受试者因无行为能力、限制行为能力，本知情同意由其监护人或者法定代理人代为签署。

受试者签名：李云峰 法定代理人签名：

日　　期：2022.4.25 日　　期：

受试者联系方式：13706530640 法定代理人联系方式：

研究者声明：

我确认已向患者解释了本研究的详细情况，特别是参加本研究可能产生的风险和收益。

研究者签名：[illegible]

日　　期：2022.4.25

研究者联系方式：13780174738

受试者签字页

受试者同意声明：

☑ 我已经阅读了上述有关本研究的介绍，且研究医生已向我详细地讲解了研究内容，在签署知情同意书前我已没有更多有关研究的疑惑需咨询。在此基础上，我自愿参加本文所介绍的临床研究，并且我的决定是基于对参加本研究可能产生的风险和受益充分了解。此外，研究者没有对我使用欺骗、利诱、胁迫等手段强行让我同意参加研究，并且我知道我可以在任何阶段无条件退出研究。

☐ 该名受试者因无行为能力、限制行为能力，本知情同意由其监护人或者法定代理人代为签署。

受试者签名：杨芸之 法定代理人签名：

日　　期：2021.10.19 日　　期：

受试者联系方式：13566146046 法定代理人联系方式：

研究者声明：

我确认已向患者解释了本研究的详细情况，特别是参加本研究可能产生的风险和收益。

研究者签名：[illegible]

日　　期：2021.10.19

研究者联系方式：13780174738

受试者签字页

受试者同意声明：

☑ 我已经阅读了上述有关本研究的介绍，且研究医生已向我详细地讲解了研究内容，在签署知情同意书前我已没有更多有关研究的疑惑需咨询。在此基础上，我自愿参加本文所介绍的临床研究，并且我的决定是基于对参加本研究可能产生的风险和受益充分了解。此外，研究者没有对我使用欺骗、利诱、胁迫等手段强行让我同意参加研究，并且我知道我可以在任何阶段无条件退出研究。

☑ 该名受试者因无行为能力、限制行为能力，本知情同意由其监护人或者法定代理人代为签署。

受试者签名：______________　　法定代理人签名：王光明

日　　期：______________　　日　　期：12.19日

受试者联系方式：______________　　法定代理人联系方式：15934719328

研究者声明：

我确认已向患者解释了本研究的详细情况，特别是参加本研究可能产生的风险和收益。

研究者签名：[illegible]

日　　期：2021.12.19

研究者联系方式：13780174738

受试者签字页

受试者同意声明：

☑ 我已经阅读了上述有关本研究的介绍，且研究医生已向我详细地讲解了研究内容，在签署知情同意书前我已没有更多有关研究的疑惑需咨询。在此基础上，我自愿参加本文所介绍的临床研究，并且我的决定是基于对参加本研究可能产生的风险和受益充分了解。此外，研究者没有对我使用欺骗、利诱、胁迫等手段强行让我同意参加研究，并且我知道我可以在任何阶段无条件退出研究。

☐ 该名受试者因无行为能力、限制行为能力，本知情同意由其监护人或者法定代理人代为签署。

受试者签名：王时中　　　　法定代理人签名：________

日　　期：2021.10.19　　　　日　　期：________

受试者联系方式：19974337862　　　　法定代理人联系方式：________

研究者声明：

我确认已向患者解释了本研究的详细情况，特别是参加本研究可能产生的风险和收益。

研究者签名：[illegible]

日　　期：2021.10.19

研究者联系方式：13780174738

受试者签字页

受试者同意声明：

☑ 我已经阅读了上述有关本研究的介绍，且研究医生已向我详细地讲解了研究内容，在签署知情同意书前我已没有更多有关研究的疑惑需咨询。在此基础上，我自愿参加本文所介绍的临床研究，并且我的决定是基于对参加本研究可能产生的风险和受益充分了解。此外，研究者没有对我使用欺骗、利诱、胁迫等手段强行让我同意参加研究，并且我知道我可以在任何阶段无条件退出研究。

☐ 该名受试者因无行为能力、限制行为能力，本知情同意由其监护人或者法定代理人代为签署。

受试者签名：金国庆　　　　法定代理人签名：________

日　　期：2021.10.27　　　　日　　期：________

受试者联系方式：13606867528　　　　法定代理人联系方式：________

研究者声明：

我确认已向患者解释了本研究的详细情况，特别是参加本研究可能产生的风险和收益。

研究者签名：[illegible]

日　　期：2021.10.27

研究者联系方式：13789174738

受试者签字页

受试者同意声明：

☑ 我已经阅读了上述有关本研究的介绍，且研究医生已向我详细地讲解了研究内容，在签署知情同意书前我已没有更多有关研究的疑惑需咨询。在此基础上，我自愿参加本文所介绍的临床研究，并且我的决定是基于对参加本研究可能产生的风险和受益充分了解。此外，研究者没有对我使用欺骗、利诱、胁迫等手段强行让我同意参加研究，并且我知道我可以在任何阶段无条件退出研究。

☐ 该名受试者因无行为能力、限制行为能力，本知情同意由其监护人或者法定代理人代为签署。

受试者签名：[illegible]　　法定代理人签名：______

日　　期：2022.3.9　　日　　期：______

受试者联系方式：13592197863　　法定代理人联系方式：______

研究者声明：

我确认已向患者解释了本研究的详细情况，特别是参加本研究可能产生的风险和收益。

研究者签名：[illegible]

日　　期：2022.3.9

研究者联系方式：13780174738

受试者签字页

受试者同意声明：

☑ 我已经阅读了上述有关本研究的介绍，且研究医生已向我详细地讲解了研究内容，在签署知情同意书前我已没有更多有关研究的疑惑需咨询。在此基础上，我自愿参加本文所介绍的临床研究，并且我的决定是基于对参加本研究可能产生的风险和受益充分了解。此外，研究者没有对我使用欺骗、利诱、胁迫等手段强行让我同意参加研究，并且我知道我可以在任何阶段无条件退出研究。

☐ 该名受试者因无行为能力、限制行为能力，本知情同意由其监护人或者法定代理人代为签署。

受试者签名：陈爱勤　　　　法定代理人签名：______

日　　期：2021.10.25　　　　日　　期：______

受试者联系方式：13736936026　　　　法定代理人联系方式：______

研究者声明：

我确认已向患者解释了本研究的详细情况，特别是参加本研究可能产生的风险和收益。

研究者签名：[illegible]

日　　期：2021.10.25

研究者联系方式：13786174738

受试者签字页

受试者同意声明：

☑ 我已经阅读了上述有关本研究的介绍，且研究医生已向我详细地讲解了研究内容，在签署知情同意书前我已没有更多有关研究的疑惑需咨询。在此基础上，我自愿参加本文所介绍的临床研究，并且我的决定是基于对参加本研究可能产生的风险和受益充分了解。此外，研究者没有对我使用欺骗、利诱、胁迫等手段强行让我同意参加研究，并且我知道我可以在任何阶段无条件退出研究。

☐ 该名受试者因无行为能力、限制行为能力，本知情同意由其监护人或者法定代理人代为签署。

受试者签名：汪新生　　法定代理人签名：________

日　　期：2021.10.22　　日　　期：________

受试者联系方式：15957578022　　法定代理人联系方式：________

研究者声明：

我确认已向患者解释了本研究的详细情况，特别是参加本研究可能产生的风险和收益。

研究者签名：[illegible]

日　　期：2021.10.22

研究者联系方式：13780174738

受试者签字页

受试者同意声明：

☑ 我已经阅读了上述有关本研究的介绍，且研究医生已向我详细地讲解了研究内容，在签署知情同意书前我已没有更多有关研究的疑惑需咨询。在此基础上，我自愿参加本文所介绍的临床研究，并且我的决定是基于对参加本研究可能产生的风险和受益充分了解。此外，研究者没有对我使用欺骗、利诱、胁迫等手段强行让我同意参加研究，并且我知道我可以在任何阶段无条件退出研究。

☐ 该名受试者因无行为能力、限制行为能力，本知情同意由其监护人或者法定代理人代为签署。

受试者签名：汤惠钦　　法定代理人签名：______

日　　期：2021.9.22　　日　　期：______

受试者联系方式：13382558858　　法定代理人联系方式：______

研究者声明：

我确认已向患者解释了本研究的详细情况，特别是参加本研究可能产生的风险和收益。

研究者签名：[illegible]

日　　期：2021.9.22

研究者联系方式：13780174738

受试者签字页

受试者同意声明：

☑ 我已经阅读了上述有关本研究的介绍，且研究医生已向我详细地讲解了研究内容，在签署知情同意书前我已没有更多有关研究的疑惑需咨询。在此基础上，我自愿参加本文所介绍的临床研究，并且我的决定是基于对参加本研究可能产生的风险和受益充分了解。此外，研究者没有对我使用欺骗、利诱、胁迫等手段强行让我同意参加研究，并且我知道我可以在任何阶段无条件退出研究。

☐ 该名受试者因无行为能力、限制行为能力，本知情同意由其监护人或者法定代理人代为签署。

受试者签名：潘国业　　法定代理人签名：______

日　　期：2022.4.19　　日　　期：______

受试者联系方式：13486787082　　法定代理人联系方式：______

研究者声明：

我确认已向患者解释了本研究的详细情况，特别是参加本研究可能产生的风险和收益。

研究者签名：[illegible]

日　　期：2022.4.19

研究者联系方式：13780174738

受试者签字页

受试者同意声明：

☑ 我已经阅读了上述有关本研究的介绍，且研究医生已向我详细地讲解了研究内容，在签署知情同意书前我已没有更多有关研究的疑惑需咨询。在此基础上，我自愿参加本文所介绍的临床研究，并且我的决定是基于对参加本研究可能产生的风险和受益充分了解。此外，研究者没有对我使用欺骗、利诱、胁迫等手段强行让我同意参加研究，并且我知道我可以在任何阶段无条件退出研究。

☐ 该名受试者因无行为能力、限制行为能力，本知情同意由其监护人或者法定代理人代为签署。

受试者签名：[illegible]　　　　法定代理人签名：________

日　　期：2021.12.3　　　　日　　期：________

受试者联系方式：18969780666　　　　法定代理人联系方式：________

研究者声明：

我确认已向患者解释了本研究的详细情况，特别是参加本研究可能产生的风险和收益。

研究者签名：[illegible]

日　　期：2021.12.3

研究者联系方式：13780174738

受试者签字页

受试者同意声明：

☑ 我已经阅读了上述有关本研究的介绍，且研究医生已向我详细地讲解了研究内容，在签署知情同意书前我已没有更多有关研究的疑惑需咨询。在此基础上，我自愿参加本文所介绍的临床研究，并且我的决定是基于对参加本研究可能产生的风险和受益充分了解。此外，研究者没有对我使用欺骗、利诱、胁迫等手段强行让我同意参加研究，并且我知道我可以在任何阶段无条件退出研究。

☐ 该名受试者因无行为能力、限制行为能力，本知情同意由其监护人或者法定代理人代为签署。

受试者签名：[illegible]　　法定代理人签名：＿＿＿＿

日　　期：2021.10.22　　日　　期：＿＿＿＿

受试者联系方式：13199759881　　法定代理人联系方式：＿＿＿＿

研究者声明：

我确认已向患者解释了本研究的详细情况，特别是参加本研究可能产生的风险和收益。

研究者签名：[illegible]

日　　期：2021.10.22

研究者联系方式：13780174738

受试者签字页

受试者同意声明：

☑ 我已经阅读了上述有关本研究的介绍，且研究医生已向我详细地讲解了研究内容，在签署知情同意书前我已没有更多有关研究的疑惑需咨询。在此基础上，我自愿参加本文所介绍的临床研究，并且我的决定是基于对参加本研究可能产生的风险和受益充分了解。此外，研究者没有对我使用欺骗、利诱、胁迫等手段强行让我同意参加研究，并且我知道我可以在任何阶段无条件退出研究。

☐ 该名受试者因无行为能力、限制行为能力，本知情同意由其监护人或者法定代理人代为签署。

受试者签名：[illegible]　　法定代理人签名：

日　　期：2021.12.8　　日　　期：

受试者联系方式：18967786807　　法定代理人联系方式：

研究者声明：

我确认已向患者解释了本研究的详细情况，特别是参加本研究可能产生的风险和收益。

研究者签名：[illegible]

日　　期：2021.12.8

研究者联系方式：13780174738

受试者签字页

受试者同意声明：

☑ 我已经阅读了上述有关本研究的介绍，且研究医生已向我详细地讲解了研究内容，在签署知情同意书前我已没有更多有关研究的疑惑需咨询。在此基础上，我自愿参加本文所介绍的临床研究，并且我的决定是基于对参加本研究可能产生的风险和受益充分了解。此外，研究者没有对我使用欺骗、利诱、胁迫等手段强行让我同意参加研究，并且我知道我可以在任何阶段无条件退出研究。

☐ 该名受试者因无行为能力、限制行为能力，本知情同意由其监护人或者法定代理人代为签署。

受试者签名：安臻　　　　法定代理人签名：________

日　　期：2021.12.20　　　　日　　期：________

受试者联系方式：13867774257　　　　法定代理人联系方式：________

研究者声明：

我确认已向患者解释了本研究的详细情况，特别是参加本研究可能产生的风险和收益。

研究者签名：[illegible]

日　　期：2021.12.20

研究者联系方式：13780174738

受试者签字页

受试者同意声明：

☑ 我已经阅读了上述有关本研究的介绍，且研究医生已向我详细地讲解了研究内容，在签署知情同意书前我已没有更多有关研究的疑惑需咨询。在此基础上，我自愿参加本文所介绍的临床研究，并且我的决定是基于对参加本研究可能产生的风险和受益充分了解。此外，研究者没有对我使用欺骗、利诱、胁迫等手段强行让我同意参加研究，并且我知道我可以在任何阶段无条件退出研究。

☐ 该名受试者因无行为能力、限制行为能力，本知情同意由其监护人或者法定代理人代为签署。

受试者签名：赵富平　　法定代理人签名：＿＿＿＿＿＿

日　　期：2021.9.7　　日　　期：＿＿＿＿＿＿

受试者联系方式：18275113232　　法定代理人联系方式：＿＿＿＿＿＿

研究者声明：

我确认已向患者解释了本研究的详细情况，特别是参加本研究可能产生的风险和收益。

研究者签名：[illegible]

日　　期：2021.9.7

研究者联系方式：13780174738

受试者签字页

受试者同意声明：

☑ 我已经阅读了上述有关本研究的介绍，且研究医生已向我详细地讲解了研究内容，在签署知情同意书前我已没有更多有关研究的疑惑需咨询。在此基础上，我自愿参加本文所介绍的临床研究，并且我的决定是基于对参加本研究可能产生的风险和受益充分了解。此外，研究者没有对我使用欺骗、利诱、胁迫等手段强行让我同意参加研究，并且我知道我可以在任何阶段无条件退出研究。

☐ 该名受试者因无行为能力、限制行为能力，本知情同意由其监护人或者法定代理人代为签署。

受试者签名：吴柏美　　法定代理人签名：______

日　　期：2021.12.3　　日　　期：______

受试者联系方式：13356142382　　法定代理人联系方式：______

研究者声明：

我确认已向患者解释了本研究的详细情况，特别是参加本研究可能产生的风险和收益。

研究者签名：[illegible]

日　　期：2021.12.3

研究者联系方式：1378[illegible]74738

受试者签字页

受试者同意声明：

☑ 我已经阅读了上述有关本研究的介绍，且研究医生已向我详细地讲解了研究内容，在签署知情同意书前我已没有更多有关研究的疑惑需咨询。在此基础上，我自愿参加本文所介绍的临床研究，并且我的决定是基于对参加本研究可能产生的风险和受益充分了解。此外，研究者没有对我使用欺骗、利诱、胁迫等手段强行让我同意参加研究，并且我知道我可以在任何阶段无条件退出研究。

☐ 该名受试者因无行为能力、限制行为能力，本知情同意由其监护人或者法定代理人代为签署。

受试者签名：左福杰　　法定代理人签名：＿＿＿＿

日　　期：2022.4.20　　日　　期：＿＿＿＿

受试者联系方式：18367893952　　法定代理人联系方式：＿＿＿＿

研究者声明：

我确认已向患者解释了本研究的详细情况，特别是参加本研究可能产生的风险和收益。

研究者签名：[illegible]

日　　期：2022.4.20

研究者联系方式：13780174738

受试者签字页

受试者同意声明：

☑ 我已经阅读了上述有关本研究的介绍，且研究医生已向我详细地讲解了研究内容，在签署知情同意书前我已没有更多有关研究的疑惑需咨询。在此基础上，我自愿参加本文所介绍的临床研究，并且我的决定是基于对参加本研究可能产生的风险和受益充分了解。此外，研究者没有对我使用欺骗、利诱、胁迫等手段强行让我同意参加研究，并且我知道我可以在任何阶段无条件退出研究。

☐ 该名受试者因无行为能力、限制行为能力，本知情同意由其监护人或者法定代理人代为签署。

受试者签名：邓泰顺　　法定代理人签名：________

日　　期：2022.3.1　　日　　期：________

受试者联系方式：13867724257　　法定代理人联系方式：________

研究者声明：

我确认已向患者解释了本研究的详细情况，特别是参加本研究可能产生的风险和收益。

研究者签名：[illegible]

日　　期：2022.3.1

研究者联系方式：13780174738

受试者签字页

受试者同意声明：

☑ 我已经阅读了上述有关本研究的介绍，且研究医生已向我详细地讲解了研究内容，在签署知情同意书前我已没有更多有关研究的疑惑需咨询。在此基础上，我自愿参加本文所介绍的临床研究，并且我的决定是基于对参加本研究可能产生的风险和受益充分了解。此外，研究者没有对我使用欺骗、利诱、胁迫等手段强行让我同意参加研究，并且我知道我可以在任何阶段无条件退出研究。

☐ 该名受试者因无行为能力、限制行为能力，本知情同意由其监护人或者法定代理人代为签署。

受试者签名：文康　　　　法定代理人签名：＿＿＿＿＿＿

日　　期：2022.3.16　　　　日　　期：＿＿＿＿＿＿

受试者联系方式：13511483728　　　　法定代理人联系方式：＿＿＿＿＿＿

研究者声明：

我确认已向患者解释了本研究的详细情况，特别是参加本研究可能产生的风险和收益。

研究者签名：[illegible]

日　　期：2022.3.16

研究者联系方式：13780174738

受试者签字页

受试者同意声明：

☑ 我已经阅读了上述有关本研究的介绍，且研究医生已向我详细地讲解了研究内容，在签署知情同意书前我已没有更多有关研究的疑惑需咨询。在此基础上，我自愿参加本文所介绍的临床研究，并且我的决定是基于对参加本研究可能产生的风险和受益充分了解。此外，研究者没有对我使用欺骗、利诱、胁迫等手段强行让我同意参加研究，并且我知道我可以在任何阶段无条件退出研究。

☐ 该名受试者因无行为能力、限制行为能力，本知情同意由其监护人或者法定代理人代为签署。

受试者签名：陈依杯　　　　法定代理人签名：________

日　　期：2022.3.10　　　　日　　期：________

受试者联系方式：13917162251　　　　法定代理人联系方式：________

研究者声明：

我确认已向患者解释了本研究的详细情况，特别是参加本研究可能产生的风险和收益。

研究者签名：[illegible]

日　　期：2022.3.10

研究者联系方式：13780174738

受试者签字页

受试者同意声明：

☑ 我已经阅读了上述有关本研究的介绍，且研究医生已向我详细地讲解了研究内容，在签署知情同意书前我已没有更多有关研究的疑惑需咨询。在此基础上，我自愿参加本文所介绍的临床研究，并且我的决定是基于对参加本研究可能产生的风险和受益充分了解。此外，研究者没有对我使用欺骗、利诱、胁迫等手段强行让我同意参加研究，并且我知道我可以在任何阶段无条件退出研究。

☐ 该名受试者因无行为能力、限制行为能力，本知情同意由其监护人或者法定代理人代为签署。

受试者签名：徐小洲　　法定代理人签名：________

日　　期：2022.3.7　　日　　期：________

受试者联系方式：15968036723　　法定代理人联系方式：________

研究者声明：

我确认已向患者解释了本研究的详细情况，特别是参加本研究可能产生的风险和收益。

研究者签名：[illegible]

日　　期：2022.3.7

研究者联系方式：1380174738

受试者签字页

受试者同意声明：

☑ 我已经阅读了上述有关本研究的介绍，且研究医生已向我详细地讲解了研究内容，在签署知情同意书前我已没有更多有关研究的疑惑需咨询。在此基础上，我自愿参加本文所介绍的临床研究，并且我的决定是基于对参加本研究可能产生的风险和受益充分了解。此外，研究者没有对我使用欺骗、利诱、胁迫等手段强行让我同意参加研究，并且我知道我可以在任何阶段无条件退出研究。

☐ 该名受试者因无行为能力、限制行为能力，本知情同意由其监护人或者法定代理人代为签署。

受试者签名：叶德书　　　法定代理人签名：______________

日　　　期：2023、2.13　　　日　　　期：______________

受试者联系方式：13222911638　　　法定代理人联系方式：______________

研究者声明：

我确认已向患者解释了本研究的详细情况，特别是参加本研究可能产生的风险和收益。

研究者签名：[illegible]

日　　　期：2023.2.13

研究者联系方式：13780174738

受试者签字页

受试者同意声明：

☑ 我已经阅读了上述有关本研究的介绍，且研究医生已向我详细地讲解了研究内容，在签署知情同意书前我已没有更多有关研究的疑惑需咨询。在此基础上，我自愿参加本文所介绍的临床研究，并且我的决定是基于对参加本研究可能产生的风险和受益充分了解。此外，研究者没有对我使用欺骗、利诱、胁迫等手段强行让我同意参加研究，并且我知道我可以在任何阶段无条件退出研究。

☐ 该名受试者因无行为能力、限制行为能力，本知情同意由其监护人或者法定代理人代为签署。

受试者签名：[illegible]　　法定代理人签名：______

日　　期：2022.10.23　　日　　期：______

受试者联系方式：15858733900　　法定代理人联系方式：______

研究者声明：

我确认已向患者解释了本研究的详细情况，特别是参加本研究可能产生的风险和收益。

研究者签名：[illegible]

日　　期：2022.10.23

研究者联系方式：1378974738

受试者签字页

受试者同意声明：

☑ 我已经阅读了上述有关本研究的介绍，且研究医生已向我详细地讲解了研究内容，在签署知情同意书前我已没有更多有关研究的疑惑需咨询。在此基础上，我自愿参加本文所介绍的临床研究，并且我的决定是基于对参加本研究可能产生的风险和受益充分了解。此外，研究者没有对我使用欺骗、利诱、胁迫等手段强行让我同意参加研究，并且我知道我可以在任何阶段无条件退出研究。

☐ 该名受试者因无行为能力、限制行为能力，本知情同意由其监护人或者法定代理人代为签署。

受试者签名：张道芳　　　　法定代理人签名：________

日　　期：2022.3.6　　　　日　　期：________

受试者联系方式：15751764633　　　　法定代理人联系方式：________

研究者声明：

我确认已向患者解释了本研究的详细情况，特别是参加本研究可能产生的风险和收益。

研究者签名：[illegible]

日　　期：2022.36

研究者联系方式：13780174738

受试者签字页

受试者同意声明：

☑ 我已经阅读了上述有关本研究的介绍，且研究医生已向我详细地讲解了研究内容，在签署知情同意书前我已没有更多有关研究的疑惑需咨询。在此基础上，我自愿参加本文所介绍的临床研究，并且我的决定是基于对参加本研究可能产生的风险和受益充分了解。此外，研究者没有对我使用欺骗、利诱、胁迫等手段强行让我同意参加研究，并且我知道我可以在任何阶段无条件退出研究。

☐ 该名受试者因无行为能力、限制行为能力，本知情同意由其监护人或者法定代理人代为签署。

受试者签名：[illegible]　　法定代理人签名：________

日　　期：2022.8.4　　日　　期：________

受试者联系方式：15597396826　　法定代理人联系方式：________

研究者声明：

我确认已向患者解释了本研究的详细情况，特别是参加本研究可能产生的风险和收益。

研究者签名：[illegible]

日　　期：2022.8.4

研究者联系方式：13780174738

受试者签字页

受试者同意声明：

☑ 我已经阅读了上述有关本研究的介绍，且研究医生已向我详细地讲解了研究内容，在签署知情同意书前我已没有更多有关研究的疑惑需咨询。在此基础上，我自愿参加本文所介绍的临床研究，并且我的决定是基于对参加本研究可能产生的风险和受益充分了解。此外，研究者没有对我使用欺骗、利诱、胁迫等手段强行让我同意参加研究，并且我知道我可以在任何阶段无条件退出研究。

☐ 该名受试者因无行为能力、限制行为能力，本知情同意由其监护人或者法定代理人代为签署。

受试者签名：叶国庆　　　　法定代理人签名：________

日　　期：2021.10.30　　　　日　　期：________

受试者联系方式：13859671023　　　　法定代理人联系方式：________

研究者声明：

我确认已向患者解释了本研究的详细情况，特别是参加本研究可能产生的风险和收益。

研究者签名：[illegible]

日　　期：2021.10.30

研究者联系方式：13780174738

受试者签字页

受试者同意声明：

☑ 我已经阅读了上述有关本研究的介绍，且研究医生已向我详细地讲解了研究内容，在签署知情同意书前我已没有更多有关研究的疑惑需咨询。在此基础上，我自愿参加本文所介绍的临床研究，并且我的决定是基于对参加本研究可能产生的风险和受益充分了解。此外，研究者没有对我使用欺骗、利诱、胁迫等手段强行让我同意参加研究，并且我知道我可以在任何阶段无条件退出研究。

☐ 该名受试者因无行为能力、限制行为能力，本知情同意由其监护人或者法定代理人代为签署。

受试者签名： 徐兴华　　法定代理人签名：＿＿＿＿

日　　期： 2022.5.23　　日　　期：＿＿＿＿

受试者联系方式： 13858534448　法定代理人联系方式：＿＿＿＿

研究者声明：

我确认已向患者解释了本研究的详细情况，特别是参加本研究可能产生的风险和收益。

研究者签名： [illegible]

日　　期： 2022.5.23

研究者联系方式： 13780174738

受试者签字页

受试者同意声明：

☑ 我已经阅读了上述有关本研究的介绍，且研究医生已向我详细地讲解了研究内容，在签署知情同意书前我已没有更多有关研究的疑惑需咨询。在此基础上，我自愿参加本文所介绍的临床研究，并且我的决定是基于对参加本研究可能产生的风险和受益充分了解。此外，研究者没有对我使用欺骗、利诱、胁迫等手段强行让我同意参加研究，并且我知道我可以在任何阶段无条件退出研究。

☐ 该名受试者因无行为能力、限制行为能力，本知情同意由其监护人或者法定代理人代为签署。

受试者签名：刘群　　法定代理人签名：________

日　　期：2023.5.6　　日　　期：________

受试者联系方式：18608321012　　法定代理人联系方式：________

研究者声明：

我确认已向患者解释了本研究的详细情况，特别是参加本研究可能产生的风险和收益。

研究者签名：[illegible]

日　　期：2023.5.6

研究者联系方式：13780174738

受试者签字页

受试者同意声明：

☑ 我已经阅读了上述有关本研究的介绍，且研究医生已向我详细地讲解了研究内容，在签署知情同意书前我已没有更多有关研究的疑惑需咨询。在此基础上，我自愿参加本文所介绍的临床研究，并且我的决定是基于对参加本研究可能产生的风险和受益充分了解。此外，研究者没有对我使用欺骗、利诱、胁迫等手段强行让我同意参加研究，并且我知道我可以在任何阶段无条件退出研究。

☐ 该名受试者因无行为能力、限制行为能力，本知情同意由其监护人或者法定代理人代为签署。

受试者签名：李吉正　　法定代理人签名：________

日　　期：2022.3.16　　日　　期：________

受试者联系方式：13086815633　　法定代理人联系方式：________

研究者声明：

我确认已向患者解释了本研究的详细情况，特别是参加本研究可能产生的风险和收益。

研究者签名：[illegible]

日　　期：2022.3.16

研究者联系方式：13780174738

受试者签字页

受试者同意声明：

☑ 我已经阅读了上述有关本研究的介绍，且研究医生已向我详细地讲解了研究内容，在签署知情同意书前我已没有更多有关研究的疑惑需咨询。在此基础上，我自愿参加本文所介绍的临床研究，并且我的决定是基于对参加本研究可能产生的风险和受益充分了解。此外，研究者没有对我使用欺骗、利诱、胁迫等手段强行让我同意参加研究，并且我知道我可以在任何阶段无条件退出研究。

☐ 该名受试者因无行为能力、限制行为能力，本知情同意由其监护人或者法定代理人代为签署。

受试者签名：[illegible]　　法定代理人签名：________

日　　期：2022.3.2　　日　　期：________

受试者联系方式：13505612402　　法定代理人联系方式：________

研究者声明：

我确认已向患者解释了本研究的详细情况，特别是参加本研究可能产生的风险和收益。

研究者签名：[illegible]

日　　期：2022.3.2

研究者联系方式：13780174738

受试者签字页

受试者同意声明：

☑ 我已经阅读了上述有关本研究的介绍，且研究医生已向我详细地讲解了研究内容，在签署知情同意书前我已没有更多有关研究的疑惑需咨询。在此基础上，我自愿参加本文所介绍的临床研究，并且我的决定是基于对参加本研究可能产生的风险和受益充分了解。此外，研究者没有对我使用欺骗、利诱、胁迫等手段强行让我同意参加研究，并且我知道我可以在任何阶段无条件退出研究。

☑ 该名受试者因无行为能力、限制行为能力，本知情同意由其监护人或者法定代理人代为签署。

受试者签名：[illegible]　　法定代理人签名：[illegible]

日　　期：2021.10.13　　日　　期：2021.10.13

受试者联系方式：________　　法定代理人联系方式：1322229669[illegible]

研究者声明：

我确认已向患者解释了本研究的详细情况，特别是参加本研究可能产生的风险和收益。

研究者签名：[illegible]

日　　期：2021.10.13

研究者联系方式：13780174738

受试者签字页

受试者同意声明：

☑ 我已经阅读了上述有关本研究的介绍，且研究医生已向我详细地讲解了研究内容，在签署知情同意书前我已没有更多有关研究的疑惑需咨询。在此基础上，我自愿参加本文所介绍的临床研究，并且我的决定是基于对参加本研究可能产生的风险和受益充分了解。此外，研究者没有对我使用欺骗、利诱、胁迫等手段强行让我同意参加研究，并且我知道我可以在任何阶段无条件退出研究。

☐ 该名受试者因无行为能力、限制行为能力，本知情同意由其监护人或者法定代理人代为签署。

受试者签名：[illegible]　　法定代理人签名：________

日　　期：2022.5.16　　日　　期：________

受试者联系方式：13852458777　　法定代理人联系方式：________

研究者声明：

我确认已向患者解释了本研究的详细情况，特别是参加本研究可能产生的风险和收益。

研究者签名：[illegible]

日　　期：2022.5.16

研究者联系方式：13780174738

受试者签字页

受试者同意声明：

☑ 我已经阅读了上述有关本研究的介绍，且研究医生已向我详细地讲解了研究内容，在签署知情同意书前我已没有更多有关研究的疑惑需咨询。在此基础上，我自愿参加本文所介绍的临床研究，并且我的决定是基于对参加本研究可能产生的风险和受益充分了解。此外，研究者没有对我使用欺骗、利诱、胁迫等手段强行让我同意参加研究，并且我知道我可以在任何阶段无条件退出研究。

☐ 该名受试者因无行为能力、限制行为能力，本知情同意由其监护人或者法定代理人代为签署。

受试者签名：郑夏生　　　　法定代理人签名：________

日　　期：2022.5.16　　　　日　　期：________

受试者联系方式：13967701652　　　　法定代理人联系方式：________

研究者声明：

我确认已向患者解释了本研究的详细情况，特别是参加本研究可能产生的风险和收益。

研究者签名：[illegible]

日　　期：2022.5.16

研究者联系方式：13789174738

受试者签字页

受试者同意声明：

☑ 我已经阅读了上述有关本研究的介绍，且研究医生已向我详细地讲解了研究内容，在签署知情同意书前我已没有更多有关研究的疑惑需咨询。在此基础上，我自愿参加本文所介绍的临床研究，并且我的决定是基于对参加本研究可能产生的风险和受益充分了解。此外，研究者没有对我使用欺骗、利诱、胁迫等手段强行让我同意参加研究，并且我知道我可以在任何阶段无条件退出研究。

☐ 该名受试者因无行为能力、限制行为能力，本知情同意由其监护人或者法定代理人代为签署。

受试者签名：陈启　　法定代理人签名：________

日　　期：2022年4月2日　　日　　期：________

受试者联系方式：18958771189　　法定代理人联系方式：________

研究者声明：

我确认已向患者解释了本研究的详细情况，特别是参加本研究可能产生的风险和收益。

研究者签名：[illegible]

日　　期：2022.4.2

研究者联系方式：13780174738

受试者签字页

受试者同意声明：

☑ 我已经阅读了上述有关本研究的介绍，且研究医生已向我详细地讲解了研究内容，在签署知情同意书前我已没有更多有关研究的疑惑需咨询。在此基础上，我自愿参加本文所介绍的临床研究，并且我的决定是基于对参加本研究可能产生的风险和受益充分了解。此外，研究者没有对我使用欺骗、利诱、胁迫等手段强行让我同意参加研究，并且我知道我可以在任何阶段无条件退出研究。

☐ 该名受试者因无行为能力、限制行为能力，本知情同意由其监护人或者法定代理人代为签署。

受试者签名：付建军　　　　法定代理人签名：________

日　　期：2022年2月14日　　　　日　　期：________

受试者联系方式：15158541709　　　　法定代理人联系方式：________

研究者声明：

我确认已向患者解释了本研究的详细情况，特别是参加本研究可能产生的风险和收益。

研究者签名：[illegible]

日　　期：2022.2.14

研究者联系方式：13780174738

受试者签字页

受试者同意声明：

☑ 我已经阅读了上述有关本研究的介绍，且研究医生已向我详细地讲解了研究内容，在签署知情同意书前我已没有更多有关研究的疑惑需咨询。在此基础上，我自愿参加本文所介绍的临床研究，并且我的决定是基于对参加本研究可能产生的风险和受益充分了解。此外，研究者没有对我使用欺骗、利诱、胁迫等手段强行让我同意参加研究，并且我知道我可以在任何阶段无条件退出研究。

☐ 该名受试者因无行为能力、限制行为能力，本知情同意由其监护人或者法定代理人代为签署。

受试者签名：董良胜　　　　法定代理人签名：______

日　　期：2023.3.7　　　　日　　期：______

受试者联系方式：13867787707　　　　法定代理人联系方式：______

研究者声明：

我确认已向患者解释了本研究的详细情况，特别是参加本研究可能产生的风险和收益。

研究者签名：[illegible]

日　　期：2023.3.7

研究者联系方式：13780174738

受试者签字页

受试者同意声明：

☑ 我已经阅读了上述有关本研究的介绍，且研究医生已向我详细地讲解了研究内容，在签署知情同意书前我已没有更多有关研究的疑惑需咨询。在此基础上，我自愿参加本文所介绍的临床研究，并且我的决定是基于对参加本研究可能产生的风险和受益充分了解。此外，研究者没有对我使用欺骗、利诱、胁迫等手段强行让我同意参加研究，并且我知道我可以在任何阶段无条件退出研究。

☐ 该名受试者因无行为能力、限制行为能力，本知情同意由其监护人或者法定代理人代为签署。

受试者签名：[illegible]　　法定代理人签名：________

日　　期：2022.1.19　　日　　期：________

受试者联系方式：13058692356　　法定代理人联系方式：________

研究者声明：

我确认已向患者解释了本研究的详细情况，特别是参加本研究可能产生的风险和收益。

研究者签名：[illegible]

日　　期：2022.1.19

研究者联系方式：13780174738

受试者签字页

受试者同意声明：

☑ 我已经阅读了上述有关本研究的介绍，且研究医生已向我详细地讲解了研究内容，在签署知情同意书前我已没有更多有关研究的疑惑需咨询。在此基础上，我自愿参加本文所介绍的临床研究，并且我的决定是基于对参加本研究可能产生的风险和受益充分了解。此外，研究者没有对我使用欺骗、利诱、胁迫等手段强行让我同意参加研究，并且我知道我可以在任何阶段无条件退出研究。

☐ 该名受试者因无行为能力、限制行为能力，本知情同意由其监护人或者法定代理人代为签署。

受试者签名：[illegible]　　法定代理人签名：______

日　　期：2022.5.5　　日　　期：______

受试者联系方式：13115735836　　法定代理人联系方式：______

研究者声明：

我确认已向患者解释了本研究的详细情况，特别是参加本研究可能产生的风险和收益。

研究者签名：[illegible]

日　　期：2022.5.5

研究者联系方式：13780174738

受试者签字页

受试者同意声明：

☑ 我已经阅读了上述有关本研究的介绍，且研究医生已向我详细地讲解了研究内容，在签署知情同意书前我已没有更多有关研究的疑惑需咨询。在此基础上，我自愿参加本文所介绍的临床研究，并且我的决定是基于对参加本研究可能产生的风险和受益充分了解。此外，研究者没有对我使用欺骗、利诱、胁迫等手段强行让我同意参加研究，并且我知道我可以在任何阶段无条件退出研究。

☐ 该名受试者因无行为能力、限制行为能力，本知情同意由其监护人或者法定代理人代为签署。

受试者签名：[illegible]　　法定代理人签名：________

日　　期：2023.4.11　　日　　期：________

受试者联系方式：13567716337　　法定代理人联系方式：________

研究者声明：

我确认已向患者解释了本研究的详细情况，特别是参加本研究可能产生的风险和收益。

研究者签名：[illegible]

日　　期：2023.4.11

研究者联系方式：13780174738

受试者签字页

受试者同意声明：

☑ 我已经阅读了上述有关本研究的介绍，且研究医生已向我详细地讲解了研究内容，在签署知情同意书前我已没有更多有关研究的疑惑需咨询。在此基础上，我自愿参加本文所介绍的临床研究，并且我的决定是基于对参加本研究可能产生的风险和受益充分了解。此外，研究者没有对我使用欺骗、利诱、胁迫等手段强行让我同意参加研究，并且我知道我可以在任何阶段无条件退出研究。

☐ 该名受试者因无行为能力、限制行为能力，本知情同意由其监护人或者法定代理人代为签署。

受试者签名：张煊　　　　法定代理人签名：________

日　　期：2022.3.12　　　　日　　期：________

受试者联系方式：13806585464　　　　法定代理人联系方式：________

研究者声明：

我确认已向患者解释了本研究的详细情况，特别是参加本研究可能产生的风险和收益。

研究者签名：[illegible]

日　　期：2022.3.12

研究者联系方式：13780174738

受试者签字页

受试者同意声明：

☑ 我已经阅读了上述有关本研究的介绍，且研究医生已向我详细地讲解了研究内容，在签署知情同意书前我已没有更多有关研究的疑惑需咨询。在此基础上，我自愿参加本文所介绍的临床研究，并且我的决定是基于对参加本研究可能产生的风险和受益充分了解。此外，研究者没有对我使用欺骗、利诱、胁迫等手段强行让我同意参加研究，并且我知道我可以在任何阶段无条件退出研究。

☐ 该名受试者因无行为能力、限制行为能力，本知情同意由其监护人或者法定代理人代为签署。

受试者签名： 潘加强　　法定代理人签名： ____________

日　　期： 2022.3.2　　日　　期： ____________

受试者联系方式： 18757729789　　法定代理人联系方式： ____________

研究者声明：

我确认已向患者解释了本研究的详细情况，特别是参加本研究可能产生的风险和收益。

研究者签名： [illegible]

日　　期： 2022.3.2

研究者联系方式： 13780174738

受试者签字页

受试者同意声明：

☑ 我已经阅读了上述有关本研究的介绍，且研究医生已向我详细地讲解了研究内容，在签署知情同意书前我已没有更多有关研究的疑惑需咨询。在此基础上，我自愿参加本文所介绍的临床研究，并且我的决定是基于对参加本研究可能产生的风险和受益充分了解。此外，研究者没有对我使用欺骗、利诱、胁迫等手段强行让我同意参加研究，并且我知道我可以在任何阶段无条件退出研究。

☐ 该名受试者因无行为能力、限制行为能力，本知情同意由其监护人或者法定代理人代为签署。

受试者签名：戴雲　　　　法定代理人签名：

日　　期：2022.4.22　　　　日　　期：

受试者联系方式：18869909589　　　　法定代理人联系方式：

研究者声明：

我确认已向患者解释了本研究的详细情况，特别是参加本研究可能产生的风险和收益。

研究者签名：[illegible]

日　　期：2022.4.22

研究者联系方式：13780174738

受试者签字页

受试者同意声明：

☑ 我已经阅读了上述有关本研究的介绍，且研究医生已向我详细地讲解了研究内容，在签署知情同意书前我已没有更多有关研究的疑惑需咨询。在此基础上，我自愿参加本文所介绍的临床研究，并且我的决定是基于对参加本研究可能产生的风险和受益充分了解。此外，研究者没有对我使用欺骗、利诱、胁迫等手段强行让我同意参加研究，并且我知道我可以在任何阶段无条件退出研究。

☐ 该名受试者因无行为能力、限制行为能力，本知情同意由其监护人或者法定代理人代为签署。

受试者签名：郭成伟　　　　法定代理人签名：＿＿＿＿＿＿

日　　期：2022.4.27　　　　日　　期：＿＿＿＿＿＿

受试者联系方式：18133284890　　　　法定代理人联系方式：＿＿＿＿＿＿

研究者声明：

我确认已向患者解释了本研究的详细情况，特别是参加本研究可能产生的风险和收益。

研究者签名：[illegible]

日　　期：2022.4.27

研究者联系方式：13780174738

受试者签字页

受试者同意声明：

☑ 我已经阅读了上述有关本研究的介绍，且研究医生已向我详细地讲解了研究内容，在签署知情同意书前我已没有更多有关研究的疑惑需咨询。在此基础上，我自愿参加本文所介绍的临床研究，并且我的决定是基于对参加本研究可能产生的风险和受益充分了解。此外，研究者没有对我使用欺骗、利诱、胁迫等手段强行让我同意参加研究，并且我知道我可以在任何阶段无条件退出研究。

☐ 该名受试者因无行为能力、限制行为能力，本知情同意由其监护人或者法定代理人代为签署。

受试者签名：方爱品　　　　法定代理人签名：________

日　　期：2022.3.21　　　　日　　期：________

受试者联系方式：13185702909　　　　法定代理人联系方式：________

研究者声明：

我确认已向患者解释了本研究的详细情况，特别是参加本研究可能产生的风险和收益。

研究者签名：[illegible]

日　　期：2022.3.21

研究者联系方式：13780174738

受试者签字页

受试者同意声明：

☑ 我已经阅读了上述有关本研究的介绍，且研究医生已向我详细地讲解了研究内容，在签署知情同意书前我已没有更多有关研究的疑惑需咨询。在此基础上，我自愿参加本文所介绍的临床研究，并且我的决定是基于对参加本研究可能产生的风险和受益充分了解。此外，研究者没有对我使用欺骗、利诱、胁迫等手段强行让我同意参加研究，并且我知道我可以在任何阶段无条件退出研究。

☐ 该名受试者因无行为能力、限制行为能力，本知情同意由其监护人或者法定代理人代为签署。

受试者签名：[illegible]　　法定代理人签名：＿＿＿＿

日　　期：2022年3月14日.　　日　　期：＿＿＿＿

受试者联系方式：13732036002　　法定代理人联系方式：＿＿＿＿

研究者声明：

我确认已向患者解释了本研究的详细情况，特别是参加本研究可能产生的风险和收益。

研究者签名：[illegible]

日　　期：2022.3.14

研究者联系方式：13780174738

受试者签字页

受试者同意声明：

☑ 我已经阅读了上述有关本研究的介绍，且研究医生已向我详细地讲解了研究内容，在签署知情同意书前我已没有更多有关研究的疑惑需咨询。在此基础上，我自愿参加本文所介绍的临床研究，并且我的决定是基于对参加本研究可能产生的风险和受益充分了解。此外，研究者没有对我使用欺骗、利诱、胁迫等手段强行让我同意参加研究，并且我知道我可以在任何阶段无条件退出研究。

☐ 该名受试者因无行为能力、限制行为能力，本知情同意由其监护人或者法定代理人代为签署。

受试者签名：胡佐金　　法定代理人签名：________

日　　期：2022.7.15　　日　　期：________

受试者联系方式：15088999768　　法定代理人联系方式：________

研究者声明：

我确认已向患者解释了本研究的详细情况，特别是参加本研究可能产生的风险和收益。

研究者签名：[illegible]

日　　期：2022.7.15

研究者联系方式：13780174738

受试者签字页

受试者同意声明：

☑ 我已经阅读了上述有关本研究的介绍，且研究医生已向我详细地讲解了研究内容，在签署知情同意书前我已没有更多有关研究的疑惑需咨询。在此基础上，我自愿参加本文所介绍的临床研究，并且我的决定是基于对参加本研究可能产生的风险和受益充分了解。此外，研究者没有对我使用欺骗、利诱、胁迫等手段强行让我同意参加研究，并且我知道我可以在任何阶段无条件退出研究。

☐ 该名受试者因无行为能力、限制行为能力，本知情同意由其监护人或者法定代理人代为签署。

受试者签名：胡春叶　　法定代理人签名：＿＿＿＿

日　　期：2022年4月26日　　日　　期：＿＿＿＿

受试者联系方式：15058956551　　法定代理人联系方式：＿＿＿＿

研究者声明：

我确认已向患者解释了本研究的详细情况，特别是参加本研究可能产生的风险和收益。

研究者签名：[illegible]

日　　期：2022.4.26

研究者联系方式：13780174738

受试者签字页

受试者同意声明：

☑ 我已经阅读了上述有关本研究的介绍，且研究医生已向我详细地讲解了研究内容，在签署知情同意书前我已没有更多有关研究的疑惑需咨询。在此基础上，我自愿参加本文所介绍的临床研究，并且我的决定是基于对参加本研究可能产生的风险和受益充分了解。此外，研究者没有对我使用欺骗、利诱、胁迫等手段强行让我同意参加研究，并且我知道我可以在任何阶段无条件退出研究。

☐ 该名受试者因无行为能力、限制行为能力，本知情同意由其监护人或者法定代理人代为签署。

受试者签名：王礼英　　　　法定代理人签名：__________

日　　期：2022年3月30日　　　　日　　期：__________

受试者联系方式：15908594548　　　　法定代理人联系方式：__________

研究者声明：

我确认已向患者解释了本研究的详细情况，特别是参加本研究可能产生的风险和收益。

研究者签名：[illegible]

日　　期：2022.3.30

研究者联系方式：13780174738

受试者签字页

受试者同意声明：

☑ 我已经阅读了上述有关本研究的介绍，且研究医生已向我详细地讲解了研究内容，在签署知情同意书前我已没有更多有关研究的疑惑需咨询。在此基础上，我自愿参加本文所介绍的临床研究，并且我的决定是基于对参加本研究可能产生的风险和受益充分了解。此外，研究者没有对我使用欺骗、利诱、胁迫等手段强行让我同意参加研究，并且我知道我可以在任何阶段无条件退出研究。

☐ 该名受试者因无行为能力、限制行为能力，本知情同意由其监护人或者法定代理人代为签署。

受试者签名：黎书方　　　　法定代理人签名：________

日　　期：2022年3月23日　　　　日　　期：________

受试者联系方式：18810095699 5　　　　法定代理人联系方式：________

研究者声明：

我确认已向患者解释了本研究的详细情况，特别是参加本研究可能产生的风险和收益。

研究者签名：[illegible]

日　　期：2022.3.23

研究者联系方式：13780174738

受试者签字页

受试者同意声明：

☑ 我已经阅读了上述有关本研究的介绍，且研究医生已向我详细地讲解了研究内容，在签署知情同意书前我已没有更多有关研究的疑惑需咨询。在此基础上，我自愿参加本文所介绍的临床研究，并且我的决定是基于对参加本研究可能产生的风险和受益充分了解。此外，研究者没有对我使用欺骗、利诱、胁迫等手段强行让我同意参加研究，并且我知道我可以在任何阶段无条件退出研究。

☐ 该名受试者因无行为能力、限制行为能力，本知情同意由其监护人或者法定代理人代为签署。

受试者签名：[illegible]　　法定代理人签名：________

日　　期：2022.03.21　　日　　期：________

受试者联系方式：15088220376　　法定代理人联系方式：________

研究者声明：

我确认已向患者解释了本研究的详细情况，特别是参加本研究可能产生的风险和收益。

研究者签名：[illegible]

日　　期：2022.3.21

研究者联系方式：13780174738

受试者签字页

受试者同意声明：

☑ 我已经阅读了上述有关本研究的介绍，且研究医生已向我详细地讲解了研究内容，在签署知情同意书前我已没有更多有关研究的疑惑需咨询。在此基础上，我自愿参加本文所介绍的临床研究，并且我的决定是基于对参加本研究可能产生的风险和受益充分了解。此外，研究者没有对我使用欺骗、利诱、胁迫等手段强行让我同意参加研究，并且我知道我可以在任何阶段无条件退出研究。

☐ 该名受试者因无行为能力、限制行为能力，本知情同意由其监护人或者法定代理人代为签署。

受试者签名：周桂花　　法定代理人签名：________

日　　期：2022.08.05　　日　　期：________

受试者联系方式：1357589952　　法定代理人联系方式：________

研究者声明：

我确认已向患者解释了本研究的详细情况，特别是参加本研究可能产生的风险和收益。

研究者签名：[illegible]

日　　期：2022.8.5

研究者联系方式：13780174738

受试者签字页

受试者同意声明：

☑ 我已经阅读了上述有关本研究的介绍，且研究医生已向我详细地讲解了研究内容，在签署知情同意书前我已没有更多有关研究的疑惑需咨询。在此基础上，我自愿参加本文所介绍的临床研究，并且我的决定是基于对参加本研究可能产生的风险和受益充分了解。此外，研究者没有对我使用欺骗、利诱、胁迫等手段强行让我同意参加研究，并且我知道我可以在任何阶段无条件退出研究。

☐ 该名受试者因无行为能力、限制行为能力，本知情同意由其监护人或者法定代理人代为签署。

受试者签名： [illegible]　　法定代理人签名：＿＿＿＿＿＿

日　　期： 2022.5.31　　日　　期：＿＿＿＿＿＿

受试者联系方式： 13451519587　　法定代理人联系方式：＿＿＿＿＿＿

研究者声明：

我确认已向患者解释了本研究的详细情况，特别是参加本研究可能产生的风险和收益。

研究者签名： [illegible]

日　　期： 2022.5.31

研究者联系方式： 13780174738

受试者签字页

受试者同意声明：

☑ 我已经阅读了上述有关本研究的介绍，且研究医生已向我详细地讲解了研究内容，在签署知情同意书前我已没有更多有关研究的疑惑需咨询。在此基础上，我自愿参加本文所介绍的临床研究，并且我的决定是基于对参加本研究可能产生的风险和受益充分了解。此外，研究者没有对我使用欺骗、利诱、胁迫等手段强行让我同意参加研究，并且我知道我可以在任何阶段无条件退出研究。

☐ 该名受试者因无行为能力、限制行为能力，本知情同意由其监护人或者法定代理人代为签署。

受试者签名：李昌立　　　法定代理人签名：＿＿＿＿＿＿

日　　期：2022.9.23　　　日　　期：＿＿＿＿＿＿

受试者联系方式：13018880355　　　法定代理人联系方式：＿＿＿＿＿＿

研究者声明：

我确认已向患者解释了本研究的详细情况，特别是参加本研究可能产生的风险和收益。

研究者签名：[illegible]

日　　期：2022.9.23

研究者联系方式：13780174738
